# Non cancer causes of death after gallbladder cancer diagnosis: a population-based analysis

**DOI:** 10.1038/s41598-023-40134-4

**Published:** 2023-08-23

**Authors:** Yang Xia, Shuangshuang Lu, Chunyan Huo, Li Fan, Min Lin, Jin Huang

**Affiliations:** Department of Gastroenterology, The Affliated Changzhou No.2 People’s Hospital of Nanjing Medical University, Changzhou, China

**Keywords:** Cancer, Medical research

## Abstract

Mortality from non cancer causes in patients with gallbladder cancer (GBC) still unclear. This study evaluated the causes and risk factors of non cancer death during different follow-up periods after GBC diagnosis. Non cancer causes of death for GBC patients diagnosed between 2000 and 2017 in Surveillance, Epidemiology and End Results database were analyzed and standardized mortality rates (SMR) for each non cancer death were calculated. Predictors for non cancer death were identified through multivariate competing risk analysis. A total 11,927 GBC patients were identified for further analysis, 9393 died during follow up. The largest proportion of non cancer deaths occurred > 3 years after diagnosis (39.4%). Most common non cancer cause were cardiovascular disease (43.3%), followed by other cause of death (34.4%) and infectious diseases (8.6%). Compared with US general population, GBC patients has higher risk of death from disease of heart (SMR, 1.58; 95%CI, 1.41–1.75), septicemia (SMR,3.21; 95%CI, 2.27–4.40), diabetes mellitus (SMR,1.97; 95%CI, 1.43–2.63), alone with other causes. Non cancer causes accounted for a significant proportion of deaths during the follow-up period after GBC diagnosis. The risk of non cancer death is higher in GBC patients than in the general population. Our study provides comprehensive assessment of death from non cancer cause in GBC patients, which has important implications for health management in GBC patients.

## Introduction

Gallbladder cancer (GBC) is the most common tumor of biliary tract and the sixth most common malignant tumor of digestive system^1^.

With the advancement of medical technology in recent years, the prognosis of cancer patients has been improved, cancer-specific mortality rates are steadily declining, leading to growing population of cancer survivors^2,3^. The increasing number of cancer survivors lead to an increasing number of non cancer deaths, including deaths from non cancer comorbidities, such as cardiovascular diseases, infectious diseases, COPD and others, which posed major threat to health, survival, and quality of life in cancer patients^4^. It has been reported that non cancer comorbidities has become the leading cause of death among patients with colorectal, breast, and prostate cancers^5^, so identifying cancer patients with higher risk of non cancer death is critical and significant.

With the diversification of therapeutic measures and the improvement of public health awareness, the mortality rates of GBC patients were generally decreasing^6^, but the study about non cancer deaths in GBC patients still lacking. Understanding the causes of death in GBC patients can help prioritize the risk of death and provide ideas for reducing the burden of death. Surveillance, Epidemiology, and End Results (SEER) based research about non cancer death with high quality population cohort has important clinical value for GBC patients. Our research assessed standardized mortality rates (SMRs) and risk factors for non cancer death in GBC patients with the goal of screening out patients who needs of early diagnosis and treatment, which was helpful for oncologists to improve prognosis. We believe our analysis will helpful to provide constructive suggestions for the health management, which can prolong GBC patients’ survival time and optimize their quality of life.

## Material and method

### Data source and study population

This study was a retrospective cohort study using data from SEER database. SEER database is a population-based cancer registry sponsored by National Cancer Institute, which regularly collects demographic and clinicopathological information of cancer patients in United States. Due to the population-based program design nature of SEER database, selected data in SEER database can be used to comparison with US general population and estimating cancer incidence, mortality and survival rates^7^. This study was performed in accordance with the Declaration of Helsinki. Due to the open source nature of SEER database, ethical approval of the publicly available information provided through SEER database was not required.

### Variable

**Figure 1:**
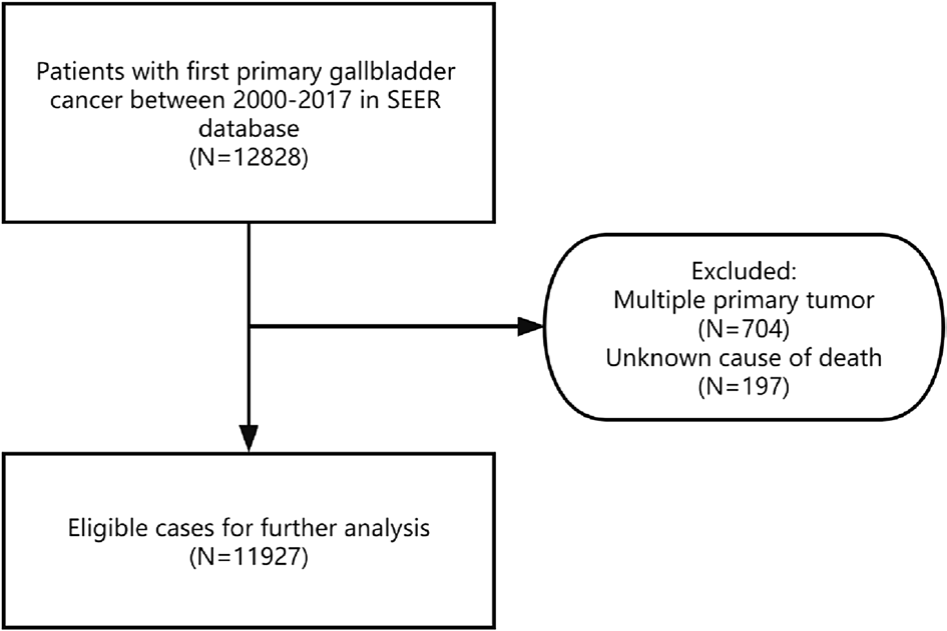
Inclusion and exclusion flowchart.

All patients diagnosed with GBC between 2000 and 2017 were extracted in SEER database through SEER*Stat software (version 8.4.0). Patients whose diagnosis was based on death certificates or autopsy reports were not included in our research. In order to reduce the interference of multiple primary cancers, we excluded patients with multiple primary cancers, in addition, unknown causes of death were also excluded, the specific screening process is shown in Fig. 1. We extracted following variable for analysis: age at diagnosis, race, sex, marital status, degree of differentiation, SEER summary stage, cause of death, surgery, radiotherapy and chemotherapy status. Well differentiated and moderately differentiated were grouped as grade I + II, poorly differentiated and undifferentiated were grouped as grade III + IV. Other race included American Indian/Alaska Native Asian or Pacific Islander patients.

### Outcome

We examined the causes of death in GBC patients, death events were grouped according to the time interval from diagnosis to death as: < 1 year, 1–3 years and > 3 years. We calculated SMR for each non cancer cause of death at different intervals after GBC diagnosis, SMR is calculated by dividing the observed number of deaths among cancer patients by the expected number of deaths among the cancer-free population, cancer-free population has similar demographic characteristic with study cohort in terms of sex, age, and race within the same interval^8^. In present study, SMR represents the risk for specific causes of death in GBC patients compared with US general population.

### Statistic analysis

We calculated SMR and corresponding 95% confidence intervals (95%CI) for non cancer deaths within different interval. Multivariate competitive risk analysis was performed to determine independent predictors for non cancer death. Hazard ratios (HRs) and 95%CI were used to represent associations between patient characteristics and cause of death. Non cancer death were defined as events of primary concern, while competitive events were defined as deaths from cancer causes. All tests were double-sided, *P* < 0.05 were considered statistically significant. Data analysis was performed by R software (version 4.0.0) and SEER*Stat software (version 8.4.0).

## Results

### Baseline characteristics

After exclusion, 11,927 GBC patients diagnosed between 2000 and 2017 were selected for further analysis. During the whole follow up periods, 9393 patients died with median survival time of 10 months (range: 0 to 215 months), the mean age of death was 71.26 years, the highest number of death occurred within 1 year, account for 62.0% of the total. Most patients in our cohort were female (70.8%), white patients account for 76.3% of the whole, black patients accounted for 12.3% and other patients accounted for 11.4%. The majority included patients in our research were well and moderate differentiation (41.0%), < 70 years old (51.1%), married (50.5%) and regional stage (35.8%). The rates of received surgery, radiotherapy and chemotherapy in our cohort were 69.9, 15.4 and 39.9%, respectively (Table 1). Concerning deaths from non cancer causes, a total of 1019 GBC patients died from non cancer causes during whole follow-up periods, the most common cause were cardiovascular disease (43.3%), followed by other cause of death (34.4%) and infectious diseases (8.6%). Compared with US general population, GBC patients has higher risk of death from disease of heart (SMR, 1.58; 95%CI, 1.41–1.75), hypertension without heart disease (SMR, 2.12; 95%CI, 1.35–3.19), septicemia (SMR, 3.21; 95%CI, 2.27–4.40), chronic liver disease and cirrhosis (SMR, 4.19; 95%CI, 2.63–6.34), nephritis, nephrotic syndrome and nephrosis (SMR, 1.65; 95%CI, 1.09–2.40) and diabetes mellitus (SMR, 1.97; 95%CI, 1.43–2.63), alone with other causes (Table 2).

**Table 1:** Baseline characteristics and distribution of survival time in patients with gallbladder cancer.

Characteristic		Total	All death	< 1 year	1–3 years	> 3 years	Total
			N(%)	Mean age	N(%)	N(%)	N(%)
Total		11,927	9393	71.26	5828	2583	982
Timing of death after diagnosis							
Age	< 70	6090	4473	60.29	2711	1337	425
	70–79	3284	2666	76.56	1681	690	295
	> = 80	2553	2254	86.74	1436	556	262
Sex	Female	8439	6625	71.55	4096	1826	703
	Male	3488	2768	70.56	1732	757	279
Race	White	9104	7207	71.98	4428	2002	777
	Black	1469	1164	67.01	745	311	108
	Other	1354	1022	71.00	655	270	97
Grade	I + II	4896	3407	72.43	1539	1252	616
	III + IV	3595	3014	70.41	2034	762	218
	Unknown	3436	2972	70.77	2255	569	148
Marital status	Single	5395	4362	73.78	2809	1092	461
	Married	6021	4643	68.92	2783	1380	480
	Unknown	511	388	70.87	236	111	41
Diagnosis year	2000–2009	6053	5347	71.85	3158	1420	769
	2010–2017	5874	4046	70.47	2670	1163	213
Stage	Localized	1212	543	77.76	184	188	171
	Regional	4269	3012	72.87	1484	1102	426
	Distant	3823	3472	67.98	2706	710	56
	Unknown	2623	2366	72.52	1454	583	329
Surgery	No	3547	3257	69.81	2672	529	56
	Yes	8333	6091	72.00	3123	2047	921
	Unknown	47	45	74.93	33	7	5
Radiotherapy	No + Unknown	10,087	7975	71.88	5207	1976	792
	Yes	1840	1418	67.77	621	607	190
Chemotherapy	No + Unknown	7170	5555	74.93	3519	1309	727
	Yes	4757	3838	65.94	2309	1274	255

**Table 2:** Standardized-mortality ratios for each cause of death following gallbladder cancer diagnosis.

Cause of death	< 1 year		1–3 years		> 3 years		Total	
	Observed	SMR(95%CI)	Observed	SMR(95%CI)	Observed	SMR(95%CI)	Observed	SMR(95%CI)
All cause of death	5828	28.37	2583	11.25	982	2.68	9393	11.71
		(27.64–29.10)		(10.82–11.70)		(2.51–2.85)		(11.48–11.95)
Non-cancer of death	355	2.21	277	1.53	387	1.31	1019	1.60
		(1.99–2.46)		(1.36–1.73)		(1.19–1.45)		(1.51–1.71)
Cardiovascular diseases	165	2.19	126	1.50	150	1.14	441	1.52
		(1.87–2.55)		(1.25–1.78)		(0.97–1.34)		(1.38–1.66)
Diseases of heart	124	2.21	104	1.66	112	1.15	340	1.58
		(1.84–2.64)		(1.36–2.02)		(0.95–1.39)		(1.41–1.75)
Hypertension without heart disease	8	3.07	6	2.00	9	1.72	23	2.12
		(1.33–6.05)		(0.73–4.36)		(0.79–3.27)		(1.35–3.19)
Aortic aneurysm and dissection	1	1.02	3	2.85	1	0.68	5	1.43
		(0.03–5.70)		(0.59–8.33)		(0.02–3.78)		(0.46–3.33)
Atherosclerosis	2	2.19	2	1.98	4	2.88	8	2.41
		(0.27–7.91)		(0.24–7.15)		(0.78–7.37)		(1.04–4.76)
Cerebrovascular diseases	28	1.99	11	0.70	23	0.93	62	1.14
		(1.32–2.88)		(0.35–1.26)		(0.59–1.40)		(0.87–1.46)
Other diseases of arteries, arterioles, capillaries	2	2.30	0	NA	1	0.67	3	0.90
		(0.28–8.32)				(0.02–3.73)		(0.19–2.63)
Infectious diseases	33	3.24	24	2.11	31	1.74	88	2.24
		(2.23–4.55)		(1.35–3.14)		(1.18–2.47)		(1.79–2.76)
Pneumonia and influenza	8	1.45	9	1.46	15	1.55	32	1.50
		(0.63–2.87)		(0.67–2.77)		(0.87–2.56)		(1.03–2.12)
Syphilis	0	NA	0	NA	0	NA	0	NA
Tuberculosis	0	NA	0	NA	0	NA	0	NA
Septicemia	20	6.51	9	2.62	9	1.69	38	3.21
		(3.97–10.05)		(1.20–4.98)		(0.77–3.20)		(2.27–4.40)
Other infectious diseases	5	3.67	6	3.92	7	2.80	18	3.34
		(1.19–8.55)		(1.44–8.54)		(1.13–5.78)		(1.98–5.28)
Respiratory diseases	18	1.44	13	0.94	24	1.08	55	1.13
		(0.86–2.28)		(0.50–1.60)		(0.69–1.60)		(0.85–1.47)
Chronic obstructive pulmonary disease and allied Cond	18	1.44	13	0.94	24	1.08	55	1.13
		(0.86–2.28)		(0.50–1.60)		(0.69–1.60)		(0.85–1.47)
Gastrointestinal diseases	8	4.51	13	6.88	6	2.25	27	4.26
		(1.95–8.89)		(3.66–11.77)		(0.82–4.89)		(2.81–6.20)
Stomach and duodenal ulcers	0	NA	4	12.52	1	2.13	5	4.62
				(3.41–32.05)		(0.05–11.89)		(1.50–10.78)
Chronic liver disease and cirrhosis	8	5.41	9	5.73	5	2.27	22	4.19
		(2.34–10.66)		(2.62–10.89)		(0.74–5.30)		(2.63–6.34)
Renal diseases	12	2.87	2	0.42	13	1.73	27	1.65
		(1.48–5.02)		(0.05–1.54)		(0.92–2.96)		(1.09–2.40)
Nephritis, nephrotic syndrome and nephrosis	12	2.87	2	0.42	13	1.73	27	1.65
		(1.48–5.02)		(0.05–1.54)		(0.92–2.96)		(1.09–2.40)
External injuries	11	1.77	9	1.31	10	0.90	30	1.24
		(0.88–3.17)		(0.60–2.48)		(0.43–1.66)		(0.84–1.77)
Accidents and adverse effects	9	1.80	7	1.25	9	0.97	25	1.26
		(0.82–3.41)		(0.50–2.57)		(0.44–1.84)		(0.81–1.85)
Suicide and self-inflicted injury	2	2.60	2	2.44	0	NA	4	1.49
		(0.31–9.39)		(0.30–8.83)				(0.40–3.80)
Homicide and legal intervention	0	NA	0	NA	1	4.77	1	1.82
						(0.12–26.57)		(0.05–10.17)
Other cause of death	108	2.14	90	1.55	153	1.50	351	1.67
		(1.76–2.59)		(1.25–1.91)		(1.27–1.75)		(1.50–1.85)
Alzheimers (ICD-9 and 10 only)	8	0.87	12	1.10	34	1.63	54	1.32
		(0.37–1.71)		(0.57–1.93)		(1.13–2.28)		(0.99–1.72)
Diabetes mellitus	14	2.28	13	1.94	18	1.80	45	1.97
		(1.25–3.83)		(1.03–3.31)		(1.06–2.84)		(1.43–2.63)
Congenital anomalies	0	NA	1	5.88	0	NA	1	1.76
				(0.15–32.74)				(0.04–9.78)
Certain conditions originating in perinatal period	0	NA	0	NA	0	NA	0	NA
Complications of pregnancy, childbirth, puerperium	0	NA	0	NA	0	NA	0	NA
Symptoms, signs and ill-defifined conditions	10	3.87	6	2.00	4	0.77	20	1.85
		(1.86–7.12)		(0.73–4.35)		(0.21–1.96)		(1.13–2.86)
Other	76	2.35	58	1.56	97	1.47	231	1.71
		(1.85–2.95)		(1.19–2.02)		(1.19–1.80)		(1.49–1.94)

NA: Not Applicable.

### Non cancer cause of death within 1 year after diagnosis

A total of 5828 GBC patients died within 1 year after diagnosis, 355 (6.1%) of them died from non cancer causes, the most common non cancer cause within 1 year were cardiovascular disease (46.5%), followed by other cause of death (30.4%) and infectious diseases (9.3%) (Table 2). Compared with US general population, GBC patients has higher risk of death from disease of heart (SMR, 2.21; 95%CI, 1.84–2.64), cerebrovascular diseases (SMR, 1.99; 95%CI, 1.32–2.88), septicemia (SMR, 6.51; 95%CI, 3.97–10.05), chronic liver disease and cirrhosis (SMR, 5.41; 95%CI, 2.34–10.66), nephritis, nephrotic syndrome and nephrosis (SMR, 2.87; 95%CI, 1.48–5.02) and diabetes mellitus (SMR, 2.28; 95%CI, 1.25–3.83), alone with other causes (Table 2). Causes of death within 1 year in specific characteristics subgroups followed the same trends with overall population. GBC patients with age < 70 years has higher risk of death from pneumonia and influenza (SMR, 9.08; 95%CI, 2.47–23.26), accidents and adverse effects (SMR, 4.93; 95%CI, 1.81–10.74) (SI Table 1). White patients has higher risk of death caused by hypertension without heart disease (SMR, 3.62; 95%CI, 1.45–7.45), cerebrovascular diseases (SMR, 1.85; 95%CI, 1.14–2.82) and septicemia (SMR, 5.02; 95%CI, 2.59–8.76) (SI Table 9). For details about all subgroups, see SI Table 1–23.

### Non cancer cause of death within 1–3 years after diagnosis

A total of 2583 GBC patients died within 1–3 years after diagnosis, 277 (10.7%) of them died from non cancer causes, most common non cancer cause within 1–3 years was cardiovascular disease (45.5%), followed by other cause of death (32.5%) and infectious diseases (8.7%) (Table 2). GBC patients has higher risk of death from disease of heart (SMR, 1.66; 95%CI, 1.36–2.02), septicemia (SMR, 2.62; 95%CI, 1.20–4.98), chronic liver disease and cirrhosis (SMR, 5.73; 95%CI, 2.62–10.89), stomach and duodenal ulcers (SMR, 12.52; 95%CI, 3.41–32.05) and diabetes mellitus (SMR, 1.94; 95%CI, 1.03–3.31) compared with US general population, alone with other causes (Table 2). Causes of death within 1–3 years after GBC diagnosis in specific subgroups followed similar trends to those in general population with the leading cause of non cancer deaths was cardiovascular disease. Female patients has higher risk of death caused by diabetes mellitus (SMR, 2.46; 95%CI, 1.23–4.41), chronic liver disease and cirrhosis (SMR, 5.57; 95%CI, 1.81–12.99) (SI Table 5). Unmarried patients has higher risk of death from pneumonia and influenza (SMR, 2.35; 95%CI, 1.02–4.64) (SI Table 7). It is worth noting that the risk of all non cancer causes of death was not significantly increased compared with US general population in age >  = 80 subgroup (SI Table 3). For details about all subgroups, see SI Table 1–23.

### Non cancer cause of death after more than 3 years after diagnosis

A total 982 GBC patients died after 3 years of diagnosis, 387 (39.4%) patients died from non cancer causes, the most common non cancer cause after 3 years of diagnosis was other cause of death (39.5%), followed by cardiovascular disease (38.8%) and infectious diseases (8.0%) (Table 2). Compared with US general population, GBC patients has higher risk of death from other infectious diseases (SMR, 2.80; 95%CI, 1.13–5.78), alzheimers (SMR, 1.63; 95%CI, 1.13–2.28) and diabetes mellitus (SMR, 1.80; 95%CI, 1.06–2.84) (Table 2). Unmarried patients has higher risk of death caused by COPD (SMR, 1.79; 95%CI, 1.04–2.86), nephritis, nephrotic syndrome and nephrosis (SMR, 2.38; 95%CI, 1.03–4.69) (SI Table 7). Married patients has higher risk of death from chronic liver disease and cirrhosis (SMR, 3.80; 95%CI, 1.23–8.86) (SI Table 6). Localized stage patients has higher risk of death from hypertension without heart disease (SMR, 4.77; 95%CI, 1.75–10.38) and pneumonia and influenza (SMR, 2.78; 95%CI, 1.02–6.04) (SI Table 13). For details about all subgroups, see SI Table 1–23.

### Risk factors for non cancer death in GBC patients

We screened the independent risk factors for non cancer death in GBC patients through multivariate competing risk analysis, the results indicated that following patient characteristics were independently associated with higher risks of non cancer death: 70–79 years old (HR: 1.835; 95% CI: 1.563–2.156), >  = 80 years old (HR: 3.021; 95% CI: 2.583–3.534), black race (HR: 1.517; 95% CI: 1.157–1.990), white race (HR: 1.309; 95% CI: 1.059–1.618) and patients who received surgery (HR: 1.499; 95% CI: 1.210–1.856). Meanwhile, the following patient characteristics were associated with lower risks of non cancer death: female sex (HR: 0.778; 95% CI: 0.677–0.894), grade III + IV (HR: 0.687; 95% CI: 0.590–0.800), regional (HR: 0.653; 95% CI: 0.552–0.772) or distant stage (HR: 0.343; 95% CI: 0.272–0.432), married patients (HR: 0.780; 95% CI: 0.679–0.895) and patients who received chemotherapy (HR: 0.567; 95% CI: 0.471–0.684). For details, see Table 3.

**Table 3:** Risk factors for non cancer death and cardiovascular death.

Characteristic		HR	95%CI	*P*
Age	< 70	Ref		
	70–79	1.835	1.563–2.156	< 0.001
	> = 80	3.021	2.583–3.534	< 0.001
Race	Other	Ref		
	Black	1.517	1.157–1.990	0.003
	White	1.309	1.059–1.618	0.013
Sex	Male	Ref		
	Female	0.778	0.677–0.894	< 0.001
Grade	I + II	Ref		
	III + IV	0.687	0.590–0.800	< 0.001
	Unknown	0.853	0.707–1.029	0.096
Stage	Localized	Ref		
	Regional	0.653	0.552–0.772	< 0.001
	Distant	0.343	0.272–0.432	< 0.001
	Unknown	0.588	0.487–0.711	< 0.001
Diagnosis year	2000–2009	Ref		
	2010–2017	0.667	0.579–0.768	< 0.001
Marital status	Single	Ref		
	Married	0.780	0.679–0.895	< 0.001
	Unknown	0.979	0.739–1.296	0.880
Surgery	No	Ref		
	Yes	1.499	1.210–1.856	< 0.001
	Unknown	0.497	0.125–1.973	0.320
Radiation	No + Unknown	Ref		
	Yes	1.076	0.864–1.339	0.510
Chemotherapy	No + Unknown	Ref		
	Yes	0.567	0.471–0.684	< 0.001

## Discussion

Our study showed that most deaths among GBC patients occurred within 1 year after diagnosis and most deaths were caused by GBC. However, the proportion of GBC related deaths decreased over time, while non cancer deaths accounted for an increasing proportion after diagnosis. Among patients who survived more than 3 years in our research, 39.4% of them died from non cancer causes. Common non cancer causes of death in GBC patients included cardiovascular disease, infectious disease and others cause of death. In our analysis, the proportion of non cancer death have changed over time, cardiovascular death (CVD) has consistently dominated.

Cancer and heart disease are two main causes of death worldwide^9^. Previous study assessing causes of death in cancer patients pointed to an increased risk of cardiovascular disease in cancer patients^10,11^. A SEER database analysis concluded that CVD in cancer patients was time-dependent, with a high risk of CVD in the first year after diagnosis^2^. Our research get the same conclusion, in our research, GBC patients has higher risk of CVD (SMR, 1.52; 95%CI, 1.38–1.66) during the entire follow-up period, especially within 1 year after diagnosis (SMR, 2.19; 95%CI, 1.87–2.55). The association between GBC and CVD may be attributable to multiple factors, cancer patients tend to have more risk factors for cardiovascular disease, based on previous research, cancer survivors were more likely than cancer-free survivors to have high blood pressure, diabetes, dyslipidemia, overweight and a history of smoking^12^. In addition, cancer patients are at risk of developing deep vein thrombosis and pulmonary embolism^13^. On the other hand, due to the psychological burden of tumor diagnosis, treatment and monitoring, additional psychological stress may be placed on patients which leading to the occurrence of cardiovascular events^14,15^. Further, GBC related treatments, such as chemotherapy, can lead to the risk of thromboembolic events which resulted ischemic heart disease as well as cerebrovascular disease^16^, radiotherapy is also cardiotoxic, about 10–30% of cancer patients treated with radiation have been reported to develop radiation-induced heart disease 5–10 years after treatment^17^. Therefore, careful evaluation should be performed before and after cancer treatments, routine cardiac imaging and serological monitoring should be considered for high-risk CVD patients^18^.

Fatal infections are one of the leading cause of death in cancer patients which be interpreted as results of the immunosuppression caused by the malignancy itself or various modern cancer treatments^19–21^. Cancer patients often has poor nutrition lead to low immunity and easy infection^22^. Based on previous study, cancer cell affects immune system through various ways^23^, the process of tumor metastasis damage immune system, which leads to the higher risk of infection^24^, further, due to the aggressive nature of tumor cells, inadequate blood supply due to rapid tumor growth can also result infection^25^. In the present study, GBC patients has higher risk of death caused by septicemia (SMR, 3.21; 95%CI, 2.27–4.40), especially within 1 year after diagnosis (SMR, 6.51; 95%CI, 3.97–10.05). The development of sepsis is often associated with cancer related treatments, both surgery and chemotherapy increase the risk of sepsis^26,27^. In addition, neutropenia as a result of antitumor therapy is a common clinical phenomenon, which has been shown to be independently associated with sepsis^28–30^. Compared with general population, cancer patients has greater risk of death from pneumonia^31^, our research get the similar conclusion in GBC patients (SMR, 1.50; 95%CI, 1.03–2.12). The susceptibility of pneumonia in cancer patients comes from several factors, including disease itself, chemotherapy and immune dysfunction^32^, it is reported that 50% of septic shock is caused by bacterial pneumonia within cancer patients^31^. Therefore, regular monitoring of infection indicators and prompt antibiotic treatment are also important parts of the treatment and follow-up strategy for GBC patients.

About 20 percent of cancer patients has diabetes^33^. Previous studies suggested that cancer patients, especially prostate, breast, and colorectal cancer patients, has higher risk of death from diabetes than general population^4^. In our research, GBC patients were more likely to died from diabetes than US general population (SMR, 1.97; 95%CI, 1.43–2.63). Cancer treatment measures can impact blood glucose, it has been suggested that higher risk of diabetes in cancer patients may be due to the processes of chemotherapy^34^. Further, the application of PD-1 in cancer therapy has become more and more widespread in recent years^35^. PD-1 inhibitors can rapidly induce severe insulin deficiency, leading to worsening of diabetes, which lead to the higher risk of death from diabetes^4^. In addition, because of the gallbladder is anatomically adjacent to the pancreas, small local metastases or compression may also affect blood glucose. Researches related to diabetes management models suggested that patients with diabetes have decreased blood glucose control, medication compliance, and self-management ability after cancer diagnosis^16^, this may lead to poor glycemic control in cancer patients with diabetes, leading to a series of complications that affect prognosis. Therefore, long-term diabetes care for GBC survivors is equally important during follow-up.

Based on Yang et al.’s analysis, cancer patients often has chronic comorbidities in the same or adjacent sites. Lung cancer patients, for example, have an increased risk of dying from respiratory diseases, gastrointestinal and liver cancer patients has increased risk of dying from digestive diseases^22^. In our research, GBC patients has higher risk of death caused from stomach and duodenal ulcers (SMR, 4.62; 95%CI, 1.50–10.78) and chronic liver disease and cirrhosis (SMR, 4.19; 95%CI, 2.63–6.34), confirming previous reports.

To screen out patients at high risk of non cancer death, we screened independent risk factors for non cancer death in GBC patients through multivariate competitive risk analysis, the results indicated that GBC patients with older age has higher risk of non cancer death. Elderly patients tend to have more comorbidities, such as hypertension, coronary heart disease and diabetes^36,37^, further, the decline of physical and physiological function in elderly patients are also related to the occurrence of non cancer death^38^. Mo et al.’s study about the risk of CVD in renal cancer patients suggested that black patients has higher risk of CVD than other races^39^, which may be due to that black patients has higher risk of venous thromboembolism^40^. Our study also indicated that black patients has higher risk of non cancer death in GBC patients. GBC patients with poorly differentiated and distant tumor stages has lower risk of non cancer death, these factors were reported to be independent risk factors for prognosis in GBC patients previously^41^. So it is possible that these patients may not live long enough to die from non cancer causes. Compared with married patients, unmarried GBC patients has higher risk of non cancer death, confirming previous reports^39,42^. The fact that marriage provides social support may explain this finding^30^. GBC patients diagnosed between 2010 and 2017 has lower risk of non cancer death than diagnosed between 2004 and 2009, possibly due to the advances in medical technology and growing emphasis on death from non cancer causes^43^.

Previous studies have shown that cancer patients received surgery will increases the risk of venous thromboembolism^44,45^. In addition, Hiong et al.’s studies indicated that cancer patients has an increased risk of postoperative sepsis, which leads to decreased cancer survival rates^26^, these factors may be explain the higher risk of non cancer death in GBC patients treated with surgery. Therefore, the risk of postoperative cardiovascular events in GBC patients should be considered, such as the prevention of acute hypertension and arrhythmia in postoperative care. On the other hand, received chemotherapy were protective factor for non cancer death in our analysis, consistent with previous studies^39,46,47^. This does not seem to fit with the common belief that chemotherapy causes cardiotoxicity and increases the risk of CVD. This may be due to that patients who receive regular chemotherapy can receive better health monitoring during treatment and receive more timely intervention when at healthy risk to avoid subsequent adverse events^47^. However, the underlying mechanism why chemotherapy improves non cancer death outcomes in GBC patients has not been clarified, and further studies still needed. To reduce the risk of non cancer death in GBC patients, we suggest that primary prevention should performed to higher risk patients. For most cancer survivors, the most effective strategies for primary prevention of non cancer death are smoking cessation, weight loss, exercise, proper diet, control of blood pressure and blood sugar, prevent atherosclerosis, etc., in addition, lifelong follow-up are also necessary, such as regular monitoring of some health indicators^48^.

There are several limitations in our analysis, first, information about non cancer comorbidities was not included in SEER database and we were unable to further explore the impact of these comorbidities on non cancer mortality in GBC patients, in addition, different treatment regimens and duration impact non cancer mortality, but the specific dose, type and duration of radiotherapy and chemotherapy regimens are not recorded in detail in SEER database^46^. Second, all patients in SEER database were selected in United States, so cases from Europe and Asia are needed to further validate our study. Finally, because the study was retrospective, which could biased the results. Despite some limitations mentioned above, this study is still meaningful and helpful in the clinical management for GBC patients.

## Conclusion

During the whole follow-up period after GBC diagnosis, the proportion of non cancer death in GBC patients gradually increased with the prolongation of diagnosis time, cardiovascular diseases and infectious diseases were the common causes. GBC patients has higher risk of death caused from cardiovascular diseases, infectious diseases, other cause of death and gastrointestinal diseases. These findings have important implications for clinical management in GBC patients.

### Supplementary Information

Supplementary Table 1.

Supplementary Table 2.

Supplementary Table 3.

Supplementary Table 4.

Supplementary Table 5.

Supplementary Table 6.

Supplementary Table 7.

Supplementary Table 8.

Supplementary Table 9.

Supplementary Table 10.

Supplementary Table 11.

Supplementary Table 12.

Supplementary Table 13.

Supplementary Table 14.

Supplementary Table 15.

Supplementary Table 16.

Supplementary Table 17.

Supplementary Table 18.

Supplementary Table 19.

Supplementary Table 20.

Supplementary Table 21.

Supplementary Table 22.

Supplementary Table 23.

## Data Availability

The datasets analyzed in this study are available in the SEER repository and can be obtained from: https://seer.cancer.gov/data/.

## Abbreviations

GBC: Gallbladder cancer
SEER: Epidemiology and end results
SMR: Standardized mortality rates; 95%
CI: 95% Confidence intervals
HRs: Hazard ratios
CVD: Cardiovascular death
