# Supplementary material for: Non cancer causes of death after gallbladder cancer diagnosis: a population-based analysis
Source: Sci Rep. 2023 Aug 23;13:13746. doi: 10.1038/s41598-023-40134-4 (PMC10447554; doi:10.1038/s41598-023-40134-4)
Supplement: Supplementary file 1 — Supplementary Table 1. [file 41598_2023_40134_MOESM1_ESM.docx]

| Cause of death | <1 year | | 1-3 years | | >3years | | Total | |
| --- | --- | --- | --- | --- | --- | --- | --- | --- |
|  | Observed | SMR(95%CI) | Observed | SMR(95%CI) | Observed | SMR(95%CI) | Observed | SMR(95%CI) |
| **ALL cause of death** | 2711 | 86.97  (83.73-90.31) | 1337 | 35.72  (33.83-37.68) | 425 | 5.02  (4.55-5.52) | 4473 | 29.17  (28.32-30.04) |
| **Non-cancer of death** | 115 | 5.72  (4.72-6.86) | 77 | 3.17  (2.50-3.96) | 90 | 1.56  (1.26-1.92) | 282 | 2.77  (2.45-3.11) |
| **Cardiovascular diseases** | 40 | 4.68  (3.35-6.38) | 29 | 2.82  (1.89-4.04) | 30 | 1.25  (0.85-1.79) | 99 | 2.32  (1.88-2.82) |
| Diseases of heart | 32 | 4.81  (3.29-6.78) | 26 | 3.26  (2.13-4.77) | 20 | 1.11  (0.68-1.71) | 78 | 2.39  (1.89-2.98) |
| Hypertension without heart disease | 1 | 3.50  (0.09-19.50) | 1 | 2.85  (0.07-16.89) | 2 | 2.24  (0.27-8.08) | 4 | 2.61  (0.71-6.69) |
| Aortic aneurysm and dissection | 1 | 7.21  (0.18-40.18) | 0 | NA | 0 | NA | 1 | 1.48  (0.04-8.23) |
| Atherosclerosis | 0 | NA | 0 | NA | 1 | 6.66  (0.17-37.12) | 1 | 3.91  (0.10-21.80) |
| Cerebrovascular diseases | 6 | 4.59  (1.68-9.99) | 2 | 1.24  (0.15-4.48) | 7 | 1.67  (0.67-3.45) | 15 | 2.11  (1.18-3.48) |
| Other diseases of arteries, arterioles, capillaries | 0 | NA | 0 | NA | 0 | NA | 0 | NA |
| **Infectious diseases** | 19 | 13.15  (7.92-20.53) | 8 | 4.66  (2.01-9.18) | 10 | 2.57  (1.23-4.73) | 37 | 5.25  (3.70-7.24) |
| Pneumonia and influenza | 4 | 9.08  (2.47-23.26) | 1 | 1.81  (0.05-10.10) | 4 | 2.69  (0.73-6.90) | 9 | 3.63  (1.66-6.90) |
| Syphilis | 0 | NA | 0 | NA | 0 | NA | 0 | NA |
| Tuberculosis | 0 | NA | 0 | NA | 0 | NA | 0 | NA |
| Septicemia | 10 | 19.54  (9.37-35.93) | 4 | 6.44  (1.75-16.49) | 1 | 0.70  (0.02-3.91) | 15 | 5.86  (3.28-9.67) |
| Other infectious diseases | 5 | 10.39  (3.37-24.26) | 3 | 5.66  (1.17-16.53) | 5 | 5.29  (1.72-12.33) | 13 | 6.64  (3.54-11.36) |
| **Respiratory diseases** | 5 | 2.83  (0.92-6.60) | 4 | 1.75  (0.48-4.49) | 8 | 1.32  (0.57-2.61) | 17 | 1.69  (0.98-2.70) |
| Chronic obstructive pulmonary disease and allied Cond | 5 | 2.83  (0.92-6.60) | 4 | 1.75  (0.48-4.49) | 8 | 1.32  (0.57-2.61) | 17 | 1.69  (0.98-2.70) |
| **Gastrointestinal diseases** | 4 | 5.37  (1.46-13.75) | 9 | 10.75  (4.92-20.42) | 4 | 2.79  (0.76-7.14) | 17 | 5.64  (3.28-9.03) |
| Stomach and duodenal ulcers | 0 | NA | 4 | 80.82  (22.02-206.93) | 0 | NA | 4 | 19.97  (5.44-51.13) |
| Chronic liver disease and cirrhosis | 4 | 5.69  (1.55-14.57) | 5 | 6.35  (2.06-14.82) | 4 | 3.02  (0.82-7.73) | 13 | 4.62  (2.46-7.90) |
| **Renal diseases** | 4 | 7.19  (1.96-18.41) | 0 | NA | 5 | 2.98  (0.97-6.95) | 9 | 3.08  (1.41-5.85) |
| Nephritis, nephrotic syndrome and nephrosis | 4 | 7.19  (1.96-18.41) | 0 | NA | 5 | 2.98  (0.97-6.95) | 9 | 3.08  (1.41-5.85) |
| **External injuries** | 7 | 3.96  (1.59-8.15) | 5 | 2.54  (0.82-5.92) | 2 | 0.57  (0.07-2.06) | 14 | 1.93  (1.06-3.24) |
| Accidents and adverse effects | 6 | 4.93  (1.81-10.74) | 3 | 2.19  (0.45-6.41) | 1 | 0.39  (0.01-2.18) | 10 | 1.94  (0.93-3.57) |
| Suicide and self-inflicted injury | 1 | 2.46  (0.06-13.72) | 2 | 4.49  (0.54-16.21) | 0 | NA | 3 | 1.96  (0.40-5.72) |
| Homicide and legal intervention | 0 | NA | 0 | NA | 1 | 6.92  (0.18-38.54) | 1 | 2.74  (0.07-15.27) |
| **Other cause of death** | 36 | 6.78  (4.75-9.39) | 22 | 3.38  (2.12-5.11) | 31 | 1.81  (1.23-2.56) | 89 | 3.07  (2.47-3.78) |
| Alzheimers (ICD-9 and 10 only) | 0 | NA | 0 | NA | 3 | 1.82  (0.38-5.33) | 3 | 1.46  (0.30-4.27) |
| Diabetes mellitus | 2 | 1.57  (0.19-5.69) | 3 | 1.98  (0.41-5.78) | 4 | 1.25  (0.34-3.21) | 9 | 1.51  (0.69-2.86) |
| Congenital anomalies | 0 | NA | 0 | NA | 0 | NA | 0 | NA |
| Certain conditions originating in perinatal period | 0 | NA | 0 | NA | 0 | NA | 0 | NA |
| Complications of pregnancy, childbirth, puerperium | 0 | NA | 0 | NA | 0 | NA | 0 | NA |
| Symptoms, signs and ill-defifined conditions | 4 | 15.72  (4.28-40.24) | 0 | NA | 0 | NA | 4 | 3.24  (0.88-8.29) |
| Other | 30 | 8.44  (5.70-12.05) | 19 | 4.35  (2.62-6.79) | 24 | 2.08  (1.33-3.09) | 73 | 3.75  (2.94-4.72) |

Additional Table 1: Standardized-mortality ratios following gallbladder cancer diagnosis in patients with age < 70.
