# Supplementary material for: Non cancer causes of death after gallbladder cancer diagnosis: a population-based analysis
Source: Sci Rep. 2023 Aug 23;13:13746. doi: 10.1038/s41598-023-40134-4 (PMC10447554; doi:10.1038/s41598-023-40134-4)
Supplement: Supplementary file 2 — Supplementary Table 2. [file 41598_2023_40134_MOESM2_ESM.docx]

| Cause of death | <1 year | | 1-3 years | | >3years | | Total | |
| --- | --- | --- | --- | --- | --- | --- | --- | --- |
|  | Observed | SMR(95%CI) | Observed | SMR(95%CI) | Observed | SMR(95%CI) | Observed | SMR(95%CI) |
| **ALL cause of death** | 1681 | 31.34  (29.86-32.88) | 690 | 10.79  (10.00-11.63) | 295 | 1.98  (1.76-2.22) | 2666 | 10.00  (9.63-10.39) |
| **Non-cancer of death** | 100 | 2.64  (2.15-3.22) | 68 | 1.47  (1.14-1.86) | 138 | 1.15  (0.97-1.36) | 306 | 1.50  (1.34-1.68) |
| **Cardiovascular diseases** | 44 | 2.55  (1.85-3.42) | 33 | 1.57  (1.08-2.21) | 58 | 1.09  (0.83-1.41) | 135 | 1.48  (1.24-1.75) |
| Diseases of heart | 33 | 2.57  (1.77-3.61) | 26 | 1.67  (1.09-2.45) | 41 | 1.05  (0.75-1.42) | 100 | 1.48  (1.21-1.80) |
| Hypertension without heart disease | 4 | 7.35  (2.00-18.83) | 3 | 4.42  (0.91-12.93) | 4 | 1.93  (0.53-4.94) | 11 | 3.34  (1.67-5.97) |
| Aortic aneurysm and dissection | 0 | NA | 2 | 5.53  (0.67-19.98) | 1 | 1.55  (0.04-8.64) | 3 | 2.27  (0.47-6.64) |
| Atherosclerosis | 0 | NA | 0 | NA | 2 | 3.74  (0.45-13.51) | 2 | 2.27  (0.27-8.19) |
| Cerebrovascular diseases | 6 | 1.88  (0.69-4.08) | 2 | 0.50  (0.06-1.82) | 10 | 0.98  (0.47-1.81) | 18 | 1.04  (0.62-1.64) |
| Other diseases of arteries, arterioles, capillaries | 1 | 4.54  (0.11-25.27) | 0 | NA | 0 | NA | 1 | 0.92  (0.02-5.11) |
| **Infectious diseases** | 8 | 3.35  (1.45-6.60) | 5 | 1.72  (0.56-4.01) | 12 | 1.69  (0.87-2.95) | 25 | 2.01  (1.30-2.97) |
| Pneumonia and influenza | 3 | 2.75  (0.57-8.03) | 1 | 0.72  (0.02-4.03) | 6 | 1.54  (0.57-3.36) | 10 | 1.57  (0.75-2.89) |
| Syphilis | 0 | NA | 0 | NA | 0 | NA | 0 | NA |
| Tuberculosis | 0 | NA | 0 | NA | 0 | NA | 0 | NA |
| Septicemia | 5 | 5.66  (1.84-13.21) | 3 | 2.87  (0.59-8.38) | 5 | 2.27  (0.74-5.29) | 13 | 3.15  (1.67-5.38) |
| Other infectious diseases | 0 | NA | 1 | 2.15  (0.05-11.97) | 1 | 1.01  (0.03-5.65) | 2 | 1.08  (0.13-3.91) |
| **Respiratory diseases** | 7 | 1.66  (0.67-3.42) | 1 | 0.20  (0.01-1.12) | 4 | 0.41  (0.11-1.05) | 12 | 0.63  (0.33-1.11) |
| Chronic obstructive pulmonary disease and allied Cond | 7 | 1.66  (0.67-3.42) | 1 | 0.20  (0.01-1.12) | 4 | 0.41  (0.11-1.05) | 12 | 0.63  (0.33-1.11) |
| **Gastrointestinal diseases** | 4 | 7.32  (1.99-18.75) | 4 | 6.89  (1.88-17.64) | 1 | 1.20  (0.03-6.70) | 9 | 4.60  (2.10-8.72) |
| Stomach and duodenal ulcers | 0 | NA | 0 | NA | 0 | NA | 0 | NA |
| Chronic liver disease and cirrhosis | 4 | 8.48  (2.31-21.72) | 4 | 8.12  (2.21-20.80) | 1 | 1.55  (0.04-8.65) | 9 | 5.60  (2.56-10.63) |
| **Renal diseases** | 4 | 3.56  (0.97-9.11) | 0 | NA | 2 | 0.63  (0.08-2.28) | 6 | 1.06  (0.39-2.31) |
| Nephritis, nephrotic syndrome and nephrosis | 4 | 3.56  (0.97-9.11) | 0 | NA | 2 | 0.63  (0.08-2.28) | 6 | 1.06  (0.39-2.31) |
| **External injuries** | 1 | 0.70  (0.02-3.88) | 2 | 1.17  (0.14-4.21) | 3 | 0.73  (0.15-2.14) | 6 | 0.83  (0.30-1.80) |
| Accidents and adverse effects | 0 | NA | 2 | 1.47  (0.18-5.30) | 3 | 0.85  (0.18-2.48) | 5 | 0.83  (0.27-1.94) |
| Suicide and self-inflicted injury | 1 | 4.92  (0.12-27.43) | 0 | NA | 0 | NA | 1 | 1.36  (0.03-7.60) |
| Homicide and legal intervention | 0 | NA | 0 | NA | 0 | NA | 0 | NA |
| **Other cause of death** | 32 | 2.93  (2.01-4.14) | 23 | 1.66  (1.05-2.49) | 58 | 1.39  (1.05-1.79) | 113 | 1.70  (1.40-2.04) |
| Alzheimers (ICD-9 and 10 only) | 3 | 2.19  (0.45-6.40) | 2 | 0.99  (0.12-3.59) | 12 | 1.38  (0.71-2.40) | 17 | 1.40  (0.82-2.25) |
| Diabetes mellitus | 4 | 2.05  (0.56-5.26) | 4 | 1.81  (0.49-4.64) | 10 | 2.47  (1.18-4.54) | 18 | 2.19  (1.30-3.47) |
| Congenital anomalies | 0 | NA | 0 | NA | 0 | NA | 0 | NA |
| Certain conditions originating in perinatal period | 0 | NA | 0 | NA | 0 | NA | 0 | NA |
| Complications of pregnancy, childbirth, puerperium | 0 | NA | 0 | NA | 0 | NA | 0 | NA |
| Symptoms, signs and ill-defifined conditions | 4 | 9.70  (2.64-24.83) | 4 | 7.52  (2.05-19.25) | 1 | 0.51  (0.01-2.85) | 9 | 3.10  (1.42-5.89) |
| Other | 21 | 2.94  (1.82-4.49) | 13 | 1.43  (0.76-2.45) | 35 | 1.29  (0.90-1.80) | 69 | 1.59  (1.24-2.02) |

Additional Table 2: Standardized-mortality ratios following gallbladder cancer diagnosis in patients with age between 70-79.
