# Supplementary material for: Non cancer causes of death after gallbladder cancer diagnosis: a population-based analysis
Source: Sci Rep. 2023 Aug 23;13:13746. doi: 10.1038/s41598-023-40134-4 (PMC10447554; doi:10.1038/s41598-023-40134-4)
Supplement: Supplementary file 3 — Supplementary Table 3. [file 41598_2023_40134_MOESM3_ESM.docx]

| Cause of death | <1 year | | 1-3 years | | >3years | | Total | |
| --- | --- | --- | --- | --- | --- | --- | --- | --- |
|  | Observed | SMR(95%CI) | Observed | SMR(95%CI) | Observed | SMR(95%CI) | Observed | SMR(95%CI) |
| **ALL cause of death** | 1436 | 11.90  (11.29-12.53) | 556 | 4.34  (3.98-4.71) | 262 | 1.97  (1.73-2.22) | 2254 | 5.90  (5.66-6.15) |
| **Non-cancer of death** | 140 | 1.37  (1.15-1.61) | 132 | 1.20  (1.00-1.42) | 159 | 1.36  (1.15-1.58) | 431 | 1.31  (1.19-1.44) |
| **Cardiovascular diseases** | 81 | 1.63  (1.30-2.03) | 64 | 1.21  (0.93-1.54) | 62 | 1.14  (0.88-1.46) | 207 | 1.32  (1.15-1.51) |
| Diseases of heart | 59 | 1.61  (1.23-2.08) | 52 | 1.33  (1.00-1.75) | 51 | 1.27  (0.95-1.68) | 162 | 1.40  (1.19-1.63) |
| Hypertension without heart disease | 3 | 1.69  (0.35-4.94) | 2 | 1.02  (0.12-3.67) | 3 | 1.32  (0.27-3.87) | 8 | 1.33  (0.57-2.62) |
| Aortic aneurysm and dissection | 0 | NA | 1 | 1.92  (0.05-10.68) | 0 | NA | 1 | 0.66  (0.02-3.70) |
| Atherosclerosis | 2 | 2.80  (0.34-10.12) | 2 | 2.64  (0.32-9.53) | 1 | 1.42  (0.04-7.90) | 5 | 2.30  (0.75-5.36) |
| Cerebrovascular diseases | 16 | 1.68  (0.96-2.72) | 7 | 0.69  (0.28-1.43) | 6 | 0.58  (0.21-1.27) | 29 | 0.97  (0.65-1.39) |
| Other diseases of arteries, arterioles, capillaries | 1 | 1.84  (0.05-10.24) | 0 | NA | 1 | 1.71  (0.04-9.53) | 2 | 1.17  (0.14-4.22) |
| **Infectious diseases** | 6 | 0.95  (0.35-2.06) | 11 | 1.63  (0.82-2.92) | 9 | 1.33  (0.61-2.52) | 26 | 1.31  (0.86-1.92) |
| Pneumonia and influenza | 1 | 0.25  (0.01-1.40) | 7 | 1.65  (0.67-3.41) | 5 | 1.16  (0.38-2.71) | 13 | 1.04  (0.55-1.78) |
| Syphilis | 0 | NA | 0 | NA | 0 | NA | 0 | NA |
| Tuberculosis | 0 | NA | 0 | NA | 0 | NA | 0 | NA |
| Septicemia | 5 | 2.98  (0.97-6.95) | 2 | 1.13  (0.14-4.09) | 3 | 1.76  (0.36-5.14) | 10 | 1.94  (0.93-3.57) |
| Other infectious diseases | 0 | NA | 2 | 2.82  (0.34-10.19) | 1 | 1.32  (0.03-7.37) | 3 | 1.41  (0.29-4.11) |
| **Respiratory diseases** | 6 | 0.93  (0.34-2.02) | 8 | 1.21  (0.52-2.38) | 12 | 1.85  (0.96-3.23) | 26 | 1.33  (0.87-1.94) |
| Chronic obstructive pulmonary disease and allied Cond | 6 | 0.93  (0.34-2.02) | 8 | 1.21  (0.52-2.38) | 12 | 1.85  (0.96-3.23) | 26 | 1.33  (0.87-1.94) |
| **Gastrointestinal diseases** | 0 | NA | 0 | NA | 1 | 2.46  (0.06-13.71) | 1 | 0.74  (0.02-4.10) |
| Stomach and duodenal ulcers | 0 | NA | 0 | NA | 1 | 5.81  (0.15-32.36) | 1 | 1.88  (0.05-10.48) |
| Chronic liver disease and cirrhosis | 0 | NA | 0 | NA | 0 | NA | 0 | NA |
| **Renal diseases** | 4 | 1.60  (0.44-4.10) | 2 | 0.75  (0.09-2.72) | 6 | 2.26  (0.83-4.91) | 12 | 1.54  (0.79-2.68) |
| Nephritis, nephrotic syndrome and nephrosis | 4 | 1.60  (0.44-4.10) | 2 | 0.75  (0.09-2.72) | 6 | 2.26  (0.83-4.91) | 12 | 1.54  (0.79-2.68) |
| **External injuries** | 3 | 1.00  (0.21-2.92) | 2 | 0.63  (0.08-2.26) | 5 | 1.44  (0.47-3.37) | 10 | 1.03  (0.50-1.90) |
| Accidents and adverse effects | 3 | 1.12  (0.23-3.27) | 2 | 0.70  (0.08-2.51) | 5 | 1.56  (0.51-3.65) | 10 | 1.14  (0.55-2.10) |
| Suicide and self-inflicted injury | 0 | NA | 0 | NA | 0 | NA | 0 | NA |
| Homicide and legal intervention | 0 | NA | 0 | NA | 0 | NA | 0 | NA |
| **Other cause of death** | 40 | 1.17  (0.84-1.59) | 45 | 1.20  (0.87-1.61) | 64 | 1.48  (1.14-1.89) | 149 | 1.30  (1.10-1.52) |
| Alzheimers (ICD-9 and 10 only) | 5 | 0.65  (0.21-1.52) | 10 | 1.16  (0.56-2.14) | 19 | 1.81  (1.09-2.83) | 34 | 1.27  (0.88-1.77) |
| Diabetes mellitus | 8 | 2.75  (1.19-5.41) | 6 | 2.01  (0.74-4.36) | 4 | 1.44  (0.39-3.68) | 18 | 2.07  (1.23-3.27) |
| Congenital anomalies | 0 | NA | 1 | 17.69  (0.45-98.58) | 0 | NA | 1 | 6.04  (0.15-33.68) |
| Certain conditions originating in perinatal period | 0 | NA | 0 | NA | 0 | NA | 0 | NA |
| Complications of pregnancy, childbirth, puerperium | 0 | NA | 0 | NA | 0 | NA | 0 | NA |
| Symptoms, signs and ill-defifined conditions | 2 | 1.04  (0.13-3.77) | 2 | 0.92  (0.11-3.33) | 3 | 1.16  (0.24-3.40) | 7 | 1.05  (0.42-2.16) |
| Other | 25 | 1.16  (0.75-1.71) | 26 | 1.10  (0.72-1.61) | 38 | 1.39  (0.99-1.91) | 89 | 1.23  (0.98-1.51) |

Additional Table 3: Standardized-mortality ratios following gallbladder cancer diagnosis in patients with age >= 80.
