# Supplementary material for: Non cancer causes of death after gallbladder cancer diagnosis: a population-based analysis
Source: Sci Rep. 2023 Aug 23;13:13746. doi: 10.1038/s41598-023-40134-4 (PMC10447554; doi:10.1038/s41598-023-40134-4)
Supplement: Supplementary file 4 — Supplementary Table 4. [file 41598_2023_40134_MOESM4_ESM.docx]

| Cause of death | <1 year | | 1-3 years | | >3years | | Total | |
| --- | --- | --- | --- | --- | --- | --- | --- | --- |
|  | Observed | SMR(95%CI) | Observed | SMR(95%CI) | Observed | SMR(95%CI) | Observed | SMR(95%CI) |
| **ALL cause of death** | 1732 | 26.10  (24.88-27.35) | 757 | 10.42  (9.69-11.19) | 279 | 2.65  (2.35-2.98) | 2768 | 11.33  (10.91-11.76) |
| **Non-cancer of death** | 119 | 2.40  (1.99-2.87) | 79 | 1.45  (1.14-1.80) | 112 | 1.39  (1.15-1.67) | 310 | 1.68  (1.50-1.87) |
| **Cardiovascular diseases** | 49 | 2.04  (1.51-2.70) | 31 | 1.19  (0.81-1.68) | 41 | 1.10  (0.79-1.50) | 121 | 1.39  (1.15-1.66) |
| Diseases of heart | 38 | 2.03  (1.44-2.78) | 28 | 1.37  (0.91-1.98) | 32 | 1.10  (0.75-1.55) | 98 | 1.44  (1.17-1.75) |
| Hypertension without heart disease | 4 | 5.94  (1.62-15.20) | 0 | NA | 3 | 2.53  (0.52-7.39) | 7 | 2.68  (1.08-5.51) |
| Aortic aneurysm and dissection | 1 | 2.51  (0.06-13.96) | 2 | 4.77  (0.58-17.24) | 1 | 1.92  (0.05-10.68) | 4 | 2.99  (0.81-7.64) |
| Atherosclerosis | 1 | 4.28  (0.11-23.84) | 0 | NA | 2 | 6.25  (0.76-22.57) | 3 | 3.73  (0.77-10.89) |
| Cerebrovascular diseases | 5 | 1.36  (0.44-3.18) | 1 | 0.25  (0.01-1.39) | 3 | 0.53  (0.11-1.55) | 9 | 0.68  (0.31-1.28) |
| Other diseases of arteries, arterioles, capillaries | 0 | NA | 0 | NA | 0 | NA | 0 | NA |
| **Infectious diseases** | 14 | 4.28  (2.34-7.18) | 9 | 2.52  (1.15-4.78) | 11 | 2.14  (1.07-3.82) | 34 | 2.83  (1.96-3.96) |
| Pneumonia and influenza | 3 | 1.71  (0.35-5.00) | 3 | 1.56  (0.32-4.55) | 4 | 1.44  (0.39-3.68) | 10 | 1.55  (0.74-2.85) |
| Syphilis | 0 | NA | 0 | NA | 0 | NA | 0 | NA |
| Tuberculosis | 0 | NA | 0 | NA | 0 | NA | 0 | NA |
| Septicemia | 8 | 8.44  (3.64-16.63) | 3 | 2.88  (0.59-8.41) | 4 | 2.65  (0.72-6.79) | 15 | 4.29  (2.40-7.07) |
| Other infectious diseases | 3 | 5.53  (1.14-16.17) | 3 | 5.18  (1.07-15.13) | 3 | 3.66  (0.75-10.69) | 9 | 4.64  (2.12-8.80) |
| **Respiratory diseases** | 7 | 1.70  (0.68-3.49) | 2 | 0.44  (0.05-1.59) | 8 | 1.22  (0.53-2.40) | 17 | 1.12  (0.65-1.79) |
| Chronic obstructive pulmonary disease and allied Cond | 7 | 1.70  (0.68-3.49) | 2 | 0.44  (0.05-1.59) | 8 | 1.22  (0.53-2.40) | 17 | 1.12  (0.65-1.79) |
| **Gastrointestinal diseases** | 3 | 4.09  (0.84-11.94) | 5 | 6.47  (2.10-15.11) | 4 | 4.00  (1.09-10.25) | 12 | 4.79  (2.47-8.37) |
| Stomach and duodenal ulcers | 0 | NA | 1 | 9.87  (0.25-55.01) | 0 | NA | 1 | 3.01  (0.08-16.80) |
| Chronic liver disease and cirrhosis | 3 | 4.70  (0.97-13.73) | 4 | 5.96  (1.62-15.26) | 4 | 4.63  (1.26-11.85) | 11 | 5.06  (2.53-9.05) |
| **Renal diseases** | 1 | 0.70  (0.02-3.89) | 0 | NA | 4 | 1.68  (0.46-4.31) | 5 | 0.93  (0.30-2.16) |
| Nephritis, nephrotic syndrome and nephrosis | 1 | 0.70  (0.02-3.89) | 0 | NA | 4 | 1.68  (0.46-4.31) | 5 | 0.93  (0.30-2.16) |
| **External injuries** | 8 | 3.09  (1.33-6.08) | 6 | 2.13  (0.78-4.63) | 6 | 1.48  (0.54-3.21) | 20 | 2.11  (1.29-3.26) |
| Accidents and adverse effects | 6 | 3.18  (1.17-6.91) | 4 | 1.93  (0.53-4.94) | 5 | 1.63  (0.53-3.80) | 15 | 2.13  (1.19-3.52) |
| Suicide and self-inflicted injury | 2 | 3.87  (0.47-13.98) | 2 | 3.62  (0.44-13.08) | 0 | NA | 4 | 2.22  (0.61-5.69) |
| Homicide and legal intervention | 0 | NA | 0 | NA | 1 | 10.46  (0.26-58.29) | 1 | 3.79  (0.10-21.11) |
| **Other cause of death** | 37 | 2.72  (1.92-3.75) | 26 | 1.70  (1.11-2.49) | 38 | 1.56  (1.11-2.14) | 101 | 1.90  (1.54-2.30) |
| Alzheimers (ICD-9 and 10 only) | 1 | 0.56  (0.01-3.12) | 4 | 1.91  (0.52-4.90) | 3 | 0.83  (0.17-2.43) | 8 | 1.07  (0.46-2.11) |
| Diabetes mellitus | 8 | 3.85  (1.66-7.58) | 2 | 0.89  (0.11-3.21) | 6 | 1.90  (0.70-4.13) | 16 | 2.14  (1.22-3.47) |
| Congenital anomalies | 0 | NA | 0 | NA | 0 | NA | 0 | NA |
| Certain conditions originating in perinatal period | 0 | NA | 0 | NA | 0 | NA | 0 | NA |
| Complications of pregnancy, childbirth, puerperium | 0 | NA | 0 | NA | 0 | NA | 0 | NA |
| Symptoms, signs and ill-defifined conditions | 4 | 6.40  (1.74-16.40) | 2 | 2.85  (0.34-10.29) | 1 | 0.91  (0.02-5.07) | 7 | 2.88  (1.16-5.94) |
| Other | 24 | 2.65  (1.70-3.94) | 18 | 1.76  (1.04-2.78) | 28 | 1.71  (1.14-2.47) | 70 | 1.96  (1.53-2.48) |

Additional Table 4: Standardized-mortality ratios following gallbladder cancer diagnosis in male patients.
