# Supplementary material for: Non cancer causes of death after gallbladder cancer diagnosis: a population-based analysis
Source: Sci Rep. 2023 Aug 23;13:13746. doi: 10.1038/s41598-023-40134-4 (PMC10447554; doi:10.1038/s41598-023-40134-4)
Supplement: Supplementary file 5 — Supplementary Table 5. [file 41598_2023_40134_MOESM5_ESM.docx]

| Cause of death | <1 year | | 1-3 years | | >3years | | Total | |
| --- | --- | --- | --- | --- | --- | --- | --- | --- |
|  | Observed | SMR(95%CI) | Observed | SMR(95%CI) | Observed | SMR(95%CI) | Observed | SMR(95%CI) |
| **ALL cause of death** | 4096 | 29.45  (28.55-30.36) | 1826 | 11.64  (11.11-12.19) | 703 | 2.69  (2.49-2.89) | 6625 | 11.88  (11.59-12.17) |
| **Non-cancer of death** | 236 | 2.13  (1.87-2.42) | 198 | 1.57  (1.36-1.81) | 275 | 1.29  (1.14-1.45) | 709 | 1.57  (1.46-1.69) |
| **Cardiovascular diseases** | 116 | 2.25  (1.86-2.70) | 95 | 1.64  (1.32-2.00) | 109 | 1.16  (0.95-1.40) | 320 | 1.57  (1.40-1.75) |
| Diseases of heart | 86 | 2.31  (1.84-2.85) | 76 | 1.81  (1.42-2.26) | 80 | 1.18  (0.93-1.46) | 242 | 1.64  (1.44-1.86) |
| Hypertension without heart disease | 4 | 2.07  (0.56-5.30) | 6 | 2.68  (0.98-5.83) | 6 | 1.48  (0.54-3.23) | 16 | 1.95  (1.11-3.16) |
| Aortic aneurysm and dissection | 0 | NA | 1 | 1.58  (0.04-8.79) | 0 | NA | 1 | 0.46  (0.01-2.57) |
| Atherosclerosis | 1 | 1.47  (0.04-8.19) | 2 | 2.63  (0.32-9.51) | 2 | 1.87  (0.23-6.75) | 5 | 1.99  (0.65-4.65) |
| Cerebrovascular diseases | 23 | 2.22  (1.41-3.33) | 10 | 0.86  (0.41-1.58) | 20 | 1.05  (0.64-1.63) | 53 | 1.29  (0.97-1.69) |
| Other diseases of arteries, arterioles, capillaries | 2 | 3.27  (0.40-11.82) | 0 | NA | 1 | 0.91  (0.02-5.06) | 3 | 1.25  (0.26-3.65) |
| **Infectious diseases** | 19 | 2.75  (1.66-4.30) | 15 | 1.93  (1.08-3.18) | 20 | 1.58  (0.97-2.44) | 54 | 1.98  (1.48-2.58) |
| Pneumonia and influenza | 5 | 1.34  (0.43-3.12) | 6 | 1.42  (0.52-3.08) | 11 | 1.60  (0.80-2.86) | 22 | 1.48  (0.93-2.24) |
| Syphilis | 0 | NA | 0 | NA | 0 | NA | 0 | NA |
| Tuberculosis | 0 | NA | 0 | NA | 0 | NA | 0 | NA |
| Septicemia | 12 | 5.64  (2.92-9.86) | 6 | 2.51  (0.92-5.46) | 5 | 1.31  (0.42-3.05) | 23 | 2.76  (1.75-4.14) |
| Other infectious diseases | 2 | 1.99  (0.24-7.21) | 3 | 2.67  (0.55-7.79) | 4 | 2.14  (0.58-5.48) | 9 | 2.25  (1.03-4.28) |
| **Respiratory diseases** | 11 | 1.32  (0.66-2.36) | 11 | 1.18  (0.59-2.11) | 16 | 1.02  (0.58-1.65) | 38 | 1.14  (0.81-1.56) |
| Chronic obstructive pulmonary disease and allied Cond | 11 | 1.32  (0.66-2.36) | 11 | 1.18  (0.59-2.11) | 16 | 1.02  (0.58-1.65) | 38 | 1.14  (0.81-1.56) |
| **Gastrointestinal diseases** | 5 | 4.82  (1.56-11.24) | 8 | 7.16  (3.09-14.12) | 2 | 1.20  (0.14-4.32) | 15 | 3.92  (2.19-6.46) |
| Stomach and duodenal ulcers | 0 | NA | 3 | 13.75  (2.83-40.17) | 1 | 2.99  (0.08-16.68) | 4 | 5.33  (1.45-13.64) |
| Chronic liver disease and cirrhosis | 5 | 5.95  (1.93-13.90) | 5 | 5.57  (1.81-12.99) | 1 | 0.75  (0.02-4.16) | 11 | 3.58  (1.78-6.40) |
| **Renal diseases** | 11 | 4.01  (2.00-7.18) | 2 | 0.64  (0.08-2.32) | 9 | 1.75  (0.80-3.33) | 22 | 2.00  (1.25-3.03) |
| Nephritis, nephrotic syndrome and nephrosis | 11 | 4.01  (2.00-7.18) | 2 | 0.64  (0.08-2.32) | 9 | 1.75  (0.80-3.33) | 22 | 2.00  (1.25-3.03) |
| **External injuries** | 3 | 0.83  (0.17-2.42) | 3 | 0.74  (0.15-2.16) | 4 | 0.57  (0.16-1.46) | 10 | 0.68  (0.33-1.25) |
| Accidents and adverse effects | 3 | 0.96  (0.20-2.81) | 3 | 0.85  (0.18-2.48) | 4 | 0.64  (0.18-1.65) | 10 | 0.78  (0.37-1.43) |
| Suicide and self-inflicted injury | 0 | NA | 0 | NA | 0 | NA | 0 | NA |
| Homicide and legal intervention | 0 | NA | 0 | NA | 0 | NA | 0 | NA |
| **Other cause of death** | 71 | 1.93  (1.51-2.43) | 64 | 1.50  (1.16-1.92) | 115 | 1.48  (1.22-1.77) | 250 | 1.59  (1.40-1.80) |
| Alzheimers (ICD-9 and 10 only) | 7 | 0.94  (0.83-1.94) | 8 | 0.91  (0.39-1.80) | 31 | 1.80  (1.22-2.55) | 46 | 1.37  (1.01-1.83) |
| Diabetes mellitus | 6 | 1.48  (0.54-3.22) | 11 | 2.46  (1.23-4.41) | 12 | 1.75  (0.90-3.05) | 29 | 1.89  (1.26-2.71) |
| Congenital anomalies | 0 | NA | 1 | 8.46  (0.21-47.16) | 0 | NA | 1 | 2.50  (0.06-13.93) |
| Certain conditions originating in perinatal period | 0 | NA | 0 | NA | 0 | NA | 0 | NA |
| Complications of pregnancy, childbirth, puerperium | 0 | NA | 0 | NA | 0 | NA | 0 | NA |
| Symptoms, signs and ill-defifined conditions | 6 | 3.07  (1.13-6.67) | 4 | 1.74  (0.47-4.45) | 3 | 0.73  (0.15-2.13) | 13 | 1.55  (0.83-2.65) |
| Other | 52 | 2.24  (1.67-2.93) | 40 | 1.49  (1.06-2.02) | 69 | 1.39  (1.09-1.77) | 161 | 1.62  (1.38-1.89) |

Additional Table 5: Standardized-mortality ratios following gallbladder cancer diagnosis in female patients.
