# Supplementary material for: Non cancer causes of death after gallbladder cancer diagnosis: a population-based analysis
Source: Sci Rep. 2023 Aug 23;13:13746. doi: 10.1038/s41598-023-40134-4 (PMC10447554; doi:10.1038/s41598-023-40134-4)
Supplement: Supplementary file 6 — Supplementary Table 6. [file 41598_2023_40134_MOESM6_ESM.docx]

| Cause of death | <1 year | | 1-3 years | | >3years | | Total | |
| --- | --- | --- | --- | --- | --- | --- | --- | --- |
|  | Observed | SMR(95%CI) | Observed | SMR(95%CI) | Observed | SMR(95%CI) | Observed | SMR(95%CI) |
| **ALL cause of death** | 2783 | 32.45  (31.26-33.68) | 1380 | 14.04  (13.31-14.81) | 480 | 2.66  (2.43-2.91) | 4643 | 12.74  (12.38-13.11) |
| **Non-cancer of death** | 144 | 2.26  (1.90-2.65) | 100 | 1.36  (1.10-1.65) | 166 | 1.18  (1.01-1.38) | 410 | 1.48  (1.34-1.63) |
| **Cardiovascular diseases** | 59 | 1.99  (1.51-2.56) | 39 | 1.15  (0.81-1.57) | 67 | 1.08  (0.84-1.37) | 165 | 1.31  (1.12-1.53) |
| Diseases of heart | 48 | 2.14  (1.58-2.83) | 32 | 1.25  (0.85-1.76) | 49 | 1.05  (0.78-1.39) | 129 | 1.36  (1.14-1.62) |
| Hypertension without heart disease | 4 | 4.26  (1.16-10.91) | 1 | 0.91  (0.02-5.06) | 3 | 1.28  (0.26-3.75) | 8 | 1.83  (0.79-3.60) |
| Aortic aneurysm and dissection | 1 | 2.19  (0.06-12.23) | 1 | 1.97  (0.05-11.00) | 1 | 1.28  (0.03-7.13) | 3 | 1.72  (0.35-5.03) |
| Atherosclerosis | 1 | 3.25  (0.08-18.11) | 1 | 2.81  (0.07-15.67) | 3 | 5.05  (1.04-14.76) | 5 | 3.98  (1.29-9.28) |
| Cerebrovascular diseases | 5 | 0.96  (0.31-2.25) | 4 | 0.67  (0.18-1.71) | 11 | 0.98  (0.49-1.76) | 20 | 0.89  (0.55-1.38) |
| Other diseases of arteries, arterioles, capillaries | 0 | NA | 0 | NA | 0 | NA | 0 | NA |
| **Infectious diseases** | 16 | 3.87  (2.21-6.29) | 8 | 1.69  (0.73-3.33) | 14 | 1.62  (0.88-2.71) | 38 | 2.17  (1.53-2.98) |
| Pneumonia and influenza | 5 | 2.37  (0.77-5.52) | 1 | 0.41  (0.01-2.28) | 7 | 1.54  (0.62-3.16) | 13 | 1.43  (0.76-2.44) |
| Syphilis | 0 | NA | 0 | NA | 0 | NA | 0 | NA |
| Tuberculosis | 0 | NA | 0 | NA | 0 | NA | 0 | NA |
| Septicemia | 7 | 5.46  (2.19-11.24) | 4 | 2.71  (0.74-6.94) | 4 | 1.50  (0.41-3.84) | 15 | 2.77  (1.55-4.56) |
| Other infectious diseases | 4 | 5.65  (1.54-14.46) | 3 | 3.78  (0.78-11.04) | 3 | 2.17  (0.45-6.34) | 10 | 3.47  (1.66-6.38) |
| **Respiratory diseases** | 5 | 0.89  (0.29-2.09) | 2 | 0.31  (0.04-1.12) | 5 | 0.43  (0.14-1.00) | 12 | 0.51  (0.26-0.89) |
| Chronic obstructive pulmonary disease and allied Cond | 5 | 0.89  (0.29-2.09) | 2 | 0.31  (0.04-1.12) | 5 | 0.43  (0.14-1.00) | 12 | 0.51  (0.26-0.89) |
| **Gastrointestinal diseases** | 5 | 5.33  (1.73-12.43) | 9 | 8.81  (4.03-16.73) | 5 | 3.23  (1.05-7.54) | 19 | 5.42  (3.26-8.46) |
| Stomach and duodenal ulcers | 0 | NA | 3 | 21.83  (4.50-63.80) | 0 | NA | 3 | 6.12  (1.26-17.87) |
| Chronic liver disease and cirrhosis | 5 | 6.13  (1.99-14.30) | 6 | 6.79  (2.49-14.78) | 5 | 3.80  (1.23-8.86) | 16 | 5.30  (3.03-8.61) |
| **Renal diseases** | 3 | 1.72  (0.35-5.03) | 0 | NA | 5 | 1.33  (0.43-3.10) | 8 | 1.06  (0.46-2.09) |
| Nephritis, nephrotic syndrome and nephrosis | 3 | 1.72  (0.35-5.03) | 0 | NA | 5 | 1.33  (0.43-3.10) | 8 | 1.06  (0.46-2.09) |
| **External injuries** | 7 | 2.37  (0.95-4.88) | 5 | 1.50  (0.49-3.51) | 3 | 0.51  (0.11-1.49) | 15 | 1.23  (0.69-2.04) |
| Accidents and adverse effects | 5 | 2.21  (0.72-5.16) | 4 | 1.56  (0.42-3.98) | 3 | 0.63  (0.13-1.84) | 12 | 1.25  (0.65-2.19) |
| Suicide and self-inflicted injury | 2 | 4.11  (0.50-14.85) | 1 | 1.91  (0.05-10.64) | 0 | NA | 3 | 1.71  (0.35-4.99) |
| Homicide and legal intervention | 0 | NA | 0 | NA | 0 | NA | 0 | NA |
| **Other cause of death** | 49 | 2.59  (1.92-3.43) | 37 | 1.66  (1.17-2.29) | 67 | 1.43  (1.11-1.82) | 153 | 1.74  (1.47-2.04) |
| Alzheimers (ICD-9 and 10 only) | 3 | 1.05  (0.22-3.08) | 8 | 2.29  (0.99-4.51) | 11 | 1.27  (0.63-2.27) | 22 | 1.47  (0.92-2.22) |
| Diabetes mellitus | 8 | 2.93  (1.26-5.77) | 6 | 1.95  (0.72-4.25) | 8 | 1.54  (0.66-3.03) | 22 | 2.00  (1.25-3.02) |
| Congenital anomalies | 0 | NA | 0 | NA | 0 | NA | 0 | NA |
| Certain conditions originating in perinatal period | 0 | NA | 0 | NA | 0 | NA | 0 | NA |
| Complications of pregnancy, childbirth, puerperium | 0 | NA | 0 | NA | 0 | NA | 0 | NA |
| Symptoms, signs and ill-defifined conditions | 2 | 2.26  (0.27-8.16) | 1 | 0.96  (0.02-5.35) | 1 | 0.45  (0.01-2.52) | 4 | 0.97  (0.26-2.48) |
| Other | 36 | 2.91  (2.04-4.03) | 22 | 1.51  (0.95-2.29) | 47 | 1.53  (1.13-2.04) | 105 | 1.82  (1.49-2.21) |

Additional Table 6: Standardized-mortality ratios following gallbladder cancer diagnosis in married patients.
