# Supplementary material for: Non cancer causes of death after gallbladder cancer diagnosis: a population-based analysis
Source: Sci Rep. 2023 Aug 23;13:13746. doi: 10.1038/s41598-023-40134-4 (PMC10447554; doi:10.1038/s41598-023-40134-4)
Supplement: Supplementary file 7 — Supplementary Table 7. [file 41598_2023_40134_MOESM7_ESM.docx]

| Cause of death | <1 year | | 1-3 years | | >3years | | Total | |
| --- | --- | --- | --- | --- | --- | --- | --- | --- |
|  | Observed | SMR(95%CI) | Observed | SMR(95%CI) | Observed | SMR(95%CI) | Observed | SMR(95%CI) |
| **ALL cause of death** | 2809 | 25.45  (24.52-26.41) | 1092 | 9.11  (8.58-9.67) | 461 | 2.75  (2.50-3.01) | 4362 | 10.96  (10.64-11.30) |
| **Non-cancer of death** | 197 | 2.21  (1.91-2.54) | 161 | 1.65  (1.40-1.92) | 199 | 1.43  (1.24-1.65) | 557 | 1.71  (1.57-1.86) |
| **Cardiovascular diseases** | 103 | 2.44  (1.99-2.96) | 80 | 1.74  (1.38-2.17) | 75 | 1.20  (0.95-1.51) | 258 | 1.71  (1.51-1.94) |
| Diseases of heart | 74 | 2.39  (1.87-3.00) | 68 | 2.02  (1.57-2.56) | 58 | 1.28  (0.97-1.65) | 200 | 1.82  (1.57-2.09) |
| Hypertension without heart disease | 4 | 2.59  (0.71-6.64) | 4 | 2.30  (0.63-5.89) | 5 | 1.91  (0.62-4.45) | 13 | 2.20  (1.17-3.76) |
| Aortic aneurysm and dissection | 0 | NA | 1 | 2.02  (0.05-11.24) | 0 | NA | 1 | 0.63  (0.02-3.50) |
| Atherosclerosis | 1 | 1.78  (0.05-9.94) | 1 | 1.66  (0.04-9.23) | 1 | 1.38  (0.03-7.70) | 3 | 1.59  (0.33-4.64) |
| Cerebrovascular diseases | 22 | 2.69  (1.68-4.07) | 6 | 0.68  (0.25-1.47) | 10 | 0.82  (0.40-1.52) | 38 | 1.30  (0.92-1.79) |
| Other diseases of arteries, arterioles, capillaries | 2 | 4.09  (0.50-14.77) | 0 | NA | 1 | 1.40  (0.04-7.80) | 3 | 1.73  (0.36-5.05) |
| **Infectious diseases** | 16 | 2.88  (1.64-4.67) | 15 | 2.48  (1.39-4.09) | 13 | 1.59  (0.84-2.71) | 44 | 2.22  (1.61-2.98) |
| Pneumonia and influenza | 3 | 0.96  (0.20-2.82) | 8 | 2.35  (1.02-4.64) | 7 | 1.53  (0.61-3.15) | 18 | 1.62  (0.96-2.56) |
| Syphilis | 0 | NA | 0 | NA | 0 | NA | 0 | NA |
| Tuberculosis | 0 | NA | 0 | NA | 0 | NA | 0 | NA |
| Septicemia | 12 | 7.26  (3.75-12.68) | 4 | 2.24  (0.61-5.73) | 3 | 1.25  (0.26-3.64) | 19 | 3.25  (1.96-5.07) |
| Other infectious diseases | 1 | 1.30  (0.03-7.26) | 3 | 3.62  (0.75-10.58) | 3 | 2.56  (0.53-7.48) | 7 | 2.53  (1.02-5.21) |
| **Respiratory diseases** | 11 | 1.74  (0.87-3.11) | 8 | 1.19  (0.51-2.34) | 17 | 1.79  (1.04-2.86) | 36 | 1.59  (1.12-2.21) |
| Chronic obstructive pulmonary disease and allied Cond | 11 | 1.74  (0.87-3.11) | 8 | 1.19  (0.51-2.34) | 17 | 1.79  (1.04-2.86) | 36 | 1.59  (1.12-2.21) |
| **Gastrointestinal diseases** | 3 | 3.96  (0.82-11.57) | 4 | 5.12  (1.39-13.10) | 1 | 1.01  (0.03-5.63) | 8 | 3.16  (1.37-6.23) |
| Stomach and duodenal ulcers | 0 | NA | 1 | 6.03  (0.15-33.60) | 1 | 4.69  (0.12-26.14) | 2 | 3.73  (0.45-13.47) |
| Chronic liver disease and cirrhosis | 3 | 5.00  (1.03-14.61) | 3 | 4.87  (1.00-14.23) | 0 | NA | 6 | 3.01  (1.10-6.55) |
| **Renal diseases** | 9 | 4.01  (1.84-7.62) | 2 | 0.82  (0.10-2.95) | 8 | 2.38  (1.03-4.69) | 19 | 2.36  (1.42-3.68) |
| Nephritis, nephrotic syndrome and nephrosis | 9 | 4.01  (1.84-7.62) | 2 | 0.82  (0.10-2.95) | 8 | 2.38  (1.03-4.69) | 19 | 2.36  (1.42-3.68) |
| **External injuries** | 4 | 1.35  (0.37-3.45) | 4 | 1.25  (0.34-3.19) | 4 | 0.87  (0.24-2.23) | 12 | 1.11  (0.58-1.94) |
| Accidents and adverse effects | 4 | 1.59  (0.43-4.06) | 3 | 1.09  (0.22-3.19) | 3 | 0.74  (0.15-2.17) | 10 | 1.07  (0.51-1.98) |
| Suicide and self-inflicted injury | 0 | NA | 1 | 3.88  (0.10-21.61) | 0 | NA | 1 | 1.24  (0.03-6.91) |
| Homicide and legal intervention | 0 | NA | 0 | NA | 1 | 13.31  (0.34-74.18) | 1 | 4.74  (0.12-26.40) |
| **Other cause of death** | 51 | 1.75  (1.30-2.30) | 48 | 1.47  (1.08-1.95) | 81 | 1.62  (1.29-2.01) | 180 | 1.61  (1.38-1.86) |
| Alzheimers (ICD-9 and 10 only) | 5 | 0.84  (0.27-1.96) | 4 | 0.59  (0.16-1.51) | 22 | 1.99  (1.24-3.01) | 31 | 1.30  (0.88-1.85) |
| Diabetes mellitus | 5 | 1.60  (0.52-3.73) | 7 | 2.11  (0.85-4.34) | 10 | 2.32  (1.11-4.27) | 22 | 2.05  (1.28-3.10) |
| Congenital anomalies | 0 | NA | 1 | 12.74  (0.32-70.96) | 0 | NA | 1 | 3.96  (0.10-22.05) |
| Certain conditions originating in perinatal period | 0 | NA | 0 | NA | 0 | NA | 0 | NA |
| Complications of pregnancy, childbirth, puerperium | 0 | NA | 0 | NA | 0 | NA | 0 | NA |
| Symptoms, signs and ill-defifined conditions | 8 | 5.09  (2.20-10.03) | 5 | 2.78  (0.90-6.48) | 3 | 1.09  (0.23-3.19) | 16 | 2.61  (1.49-4.25) |
| Other | 33 | 1.79  (1.23-2.51) | 31 | 1.50  (1.02-2.13) | 46 | 1.45  (1.06-1.93) | 110 | 1.55  (1.28-1.87) |

Additional Table 7: Standardized-mortality ratios following gallbladder cancer diagnosis in unmarried patients.
