# Supplementary material for: Non cancer causes of death after gallbladder cancer diagnosis: a population-based analysis
Source: Sci Rep. 2023 Aug 23;13:13746. doi: 10.1038/s41598-023-40134-4 (PMC10447554; doi:10.1038/s41598-023-40134-4)
Supplement: Supplementary file 8 — Supplementary Table 8. [file 41598_2023_40134_MOESM8_ESM.docx]

| Cause of death | <1 year | | 1-3 years | | >3years | | Total | |
| --- | --- | --- | --- | --- | --- | --- | --- | --- |
|  | Observed | SMR(95%CI) | Observed | SMR(95%CI) | Observed | SMR(95%CI) | Observed | SMR(95%CI) |
| **ALL cause of death** | 745 | 35.52  (33.01-38.16) | 311 | 13.28  (11.84-14.84) | 108 | 3.98  (3.26-4.80) | 1164 | 16.27  (15.35-17.23) |
| **Non-cancer of death** | 51 | 3.19  (2.38-4.20) | 29 | 1.60  (1.07-2.31) | 39 | 1.87  (1.33-2.56) | 119 | 2.17  (1.80-2.60) |
| **Cardiovascular diseases** | 22 | 2.79  (1.75-4.23) | 10 | 1.13  (0.54-2.07) | 16 | 1.62  (0.93-2.63) | 48 | 1.80  (1.33-2.39) |
| Diseases of heart | 18 | 3.13  (1.85-4.95) | 10 | 1.55  (0.74-2.85) | 12 | 1.67  (0.86-2.92) | 40 | 2.06  (1.47-2.81) |
| Hypertension without heart disease | 0 | NA | 0 | NA | 3 | 5.24  (1.08-15.33) | 3 | 2.02  (0.42-5.92) |
| Aortic aneurysm and dissection | 0 | NA | 0 | NA | 0 | NA | 0 | NA |
| Atherosclerosis | 0 | NA | 0 | NA | 0 | NA | 0 | NA |
| Cerebrovascular diseases | 4 | 2.74  (0.75-7.03) | 0 | NA | 1 | 0.54  (0.01-3.03) | 5 | 1.01  (0.33-2.36) |
| Other diseases of arteries, arterioles, capillaries | 0 | NA | 0 | NA | 0 | NA | 0 | NA |
| **Infectious diseases** | 9 | 7.46  (3.41-14.17) | 5 | 3.72  (1.21-8.69) | 2 | 1.32  (0.16-4.78) | 16 | 3.94  (2.25-6.40) |
| Pneumonia and influenza | 1 | 2.25  (0.06-12.52) | 2 | 3.86  (0.47-13.94) | 1 | 1.77  (0.04-9.84) | 4 | 2.61  (0.71-6.69) |
| Syphilis | 0 | NA | 0 | NA | 0 | NA | 0 | NA |
| Tuberculosis | 0 | NA | 0 | NA | 0 | NA | 0 | NA |
| Septicemia | 7 | 14.16  (5.69-29.18) | 1 | 1.81  (0.05-10.08) | 1 | 1.59  (0.04-8.84) | 9 | 5.37  (2.45-10.19) |
| Other infectious diseases | 1 | 3.89  (0.10-21.66) | 2 | 7.62  (0.92-27.53) | 0 | NA | 3 | 3.64  (0.75-10.62) |
| **Respiratory diseases** | 2 | 2.73  (0.33-9.88) | 1 | 1.23  (0.03-6.84) | 2 | 1.99  (0.24-7.19) | 5 | 1.96  (0.64-4.57) |
| Chronic obstructive pulmonary disease and allied Cond | 2 | 2.73  (0.33-9.88) | 1 | 1.23  (0.03-6.84) | 2 | 1.99  (0.24-7.19) | 5 | 1.96  (0.64-4.57) |
| **Gastrointestinal diseases** | 1 | 6.38  (0.16-35.52) | 0 | NA | 0 | NA | 1 | 1.99 |
| Stomach and duodenal ulcers | 0 | NA | 0 | NA | 0 | NA | 0 | NA |
| Chronic liver disease and cirrhosis | 1 | 7.44  (0.19-41.17) | 0 | NA | 0 | NA | 1 | 2.33  (0.06-12.97) |
| **Renal diseases** | 3 | 4.33  (0.89-12.66) | 0 | NA | 1 | 1.10  (0.03-6.11) | 4 | 1.68  (0.46-4.30) |
| Nephritis, nephrotic syndrome and nephrosis | 3 | 4.33  (0.89-12.66) | 0 | NA | 1 | 1.10  (0.03-6.11) | 4 | 1.68  (0.46-4.30) |
| **External injuries** | 2 | 3.88  (0.47-14.02) | 1 | 1.84  (0.05-10.24) | 1 | 1.61  (0.04-8.97) | 4 | 2.38  (0.65-6.09) |
| Accidents and adverse effects | 2 | 4.90  (0.59-17.69) | 1 | 2.28  (0.06-12.73) | 1 | 1.98  (0.05-11.04) | 4 | 2.96  (0.81-7.58) |
| Suicide and self-inflicted injury | 0 | NA | 0 | NA | 0 | NA | 0 | NA |
| Homicide and legal intervention | 0 | NA | 0 | NA | 0 | NA | 0 | NA |
| **Other cause of death** | 12 | 2.50  (1.29-4.36) | 12 | 2.15  (1.11-3.76) | 17 | 2.52  (1.47-4.04) | 41 | 2.40  (1.72-3.25) |
| Alzheimers (ICD-9 and 10 only) | 0 | NA | 0 | NA | 3 | 3.17  (0.65-9.27) | 3 | 1.31  (0.27-3.83) |
| Diabetes mellitus | 3 | 2.92  (0.60-8.52) | 2 | 1.79  (0.22-6.46) | 2 | 1.56  (0.19-5.63) | 7 | 2.04  (0.82-4.20) |
| Congenital anomalies | 0 | NA | 0 | NA | 0 | NA | 0 | NA |
| Certain conditions originating in perinatal period | 0 | NA | 0 | NA | 0 | NA | 0 | NA |
| Complications of pregnancy, childbirth, puerperium | 0 | NA | 0 | NA | 0 | NA | 0 | NA |
| Symptoms, signs and ill-defifined conditions | 0 | NA | 0 | NA | 0 | NA | 0 | NA |
| Other | 9 | 3.07  (1.40-5.83) | 10 | 2.94  (1.41-5.41) | 12 | 2.88  (1.49-5.04) | 31 | 2.96  (2.01-4.20) |

Additional Table 8: Standardized-mortality ratios following gallbladder cancer diagnosis in black patients.
