# Supplementary material for: Non cancer causes of death after gallbladder cancer diagnosis: a population-based analysis
Source: Sci Rep. 2023 Aug 23;13:13746. doi: 10.1038/s41598-023-40134-4 (PMC10447554; doi:10.1038/s41598-023-40134-4)
Supplement: Supplementary file 9 — Supplementary Table 9. [file 41598_2023_40134_MOESM9_ESM.docx]

| Cause of death | <1 year | | 1-3 years | | >3years | | Total | |
| --- | --- | --- | --- | --- | --- | --- | --- | --- |
|  | Observed | SMR(95%CI) | Observed | SMR(95%CI) | Observed | SMR(95%CI) | Observed | SMR(95%CI) |
| **ALL cause of death** | 4428 | 25.99  (25.23-26.77) | 2002 | 10.47  (10.02-10.94) | 777 | 2.51  (2.34-2.69) | 7207 | 10.74  (10.49-10.99) |
| **Non-cancer of death** | 279 | 2.09  (1.85-2.35) | 225 | 1.49  (1.30-1.70) | 308 | 1.23  (1.10-1.38) | 812 | 1.52  (1.42-1.63) |
| **Cardiovascular diseases** | 131 | 2.10  (1.76-2.50) | 106 | 1.52  (1.24-1.84) | 121 | 1.10  (0.91-1.31) | 358 | 1.48  (1.33-1.64) |
| Diseases of heart | 98 | 2.10  (1.71-2.56) | 87 | 1.66  (1.33-2.05) | 91 | 1.10  (0.89-1.36) | 276 | 1.52  (1.35-1.71) |
| Hypertension without heart disease | 7 | 3.62  (1.45-7.45) | 5 | 2.24  (0.73-5.22) | 5 | 1.24  (0.40-2.90) | 17 | 2.08  (1.21-3.32) |
| Aortic aneurysm and dissection | 1 | 1.22  (0.03-6.80) | 3 | 3.37  (0.70-9.86) | 1 | 0.81  (0.02-4.49) | 5 | 1.69  (0.55-3.95) |
| Atherosclerosis | 2 | 2.48  (0.30-8.97) | 2 | 2.24  (0.27-8.10) | 4 | 3.24  (0.88-8.29) | 8 | 2.73  (1.18-5.38) |
| Cerebrovascular diseases | 21 | 1.85  (1.14-2.82) | 9 | 0.71  (0.32-1.34) | 19 | 0.94  (0.56-1.46) | 49 | 1.10  (0.82-1.46) |
| Other diseases of arteries, arterioles, capillaries | 2 | 2.81  (0.34-10.15) | 0 | NA | 1 | 0.79  (0.02-4.43) | 3 | 1.08  (0.22-3.17) |
| **Infectious diseases** | 20 | 2.47  (1.51-3.82) | 17 | 1.87  (1.09-2.99) | 24 | 1.67  (1.07-2.48) | 61 | 1.93  (1.48-2.48) |
| Pneumonia and influenza | 4 | 0.89  (0.24-2.27) | 7 | 1.38  (0.55-2.84) | 10 | 1.26  (0.61-2.32) | 21 | 1.20  (0.74-1.83) |
| Syphilis | 0 | NA | 0 | NA | 0 | NA | 0 | NA |
| Tuberculosis | 0 | NA | 0 | NA | 0 | NA | 0 | NA |
| Septicemia | 12 | 5.02  (2.59-8.76) | 8 | 2.98  (1.29-5.88) | 7 | 1.62  (0.65-3.35) | 27 | 2.88  (1.90-4.19) |
| Other infectious diseases | 4 | 3.46  (0.94-8.85) | 2 | 1.54  (0.19-5.55) | 7 | 3.29  (1.32-6.79) | 13 | 2.84  (1.51-4.85) |
| **Respiratory diseases** | 16 | 1.43  (0.82-2.33) | 11 | 0.88  (0.44-1.58) | 19 | 0.95  (0.57-1.48) | 46 | 1.05  (0.77-1.41) |
| Chronic obstructive pulmonary disease and allied Cond | 16 | 1.43  (0.82-2.33) | 11 | 0.88  (0.44-1.58) | 19 | 0.95  (0.57-1.48) | 46 | 1.05  (0.77-1.41) |
| **Gastrointestinal diseases** | 6 | 4.12  (1.51-8.97) | 12 | 7.69  (3.97-13.43) | 6 | 2.73  (1.00-5.93) | 24 | 4.60  (2.95-6.84) |
| Stomach and duodenal ulcers | 0 | NA | 4 | 15.19  (4.14-38.90) | 1 | 2.61  (0.07-14.55) | 5 | 5.64  (1.83-13.16) |
| Chronic liver disease and cirrhosis | 6 | 4.94  (1.81-10.74) | 8 | 6.17  (2.66-12.15) | 5 | 2.75  (0.89-6.42) | 19 | 4.39  (2.64-6.85) |
| **Renal diseases** | 9 | 2.85  (1.30-5.40) | 2 | 0.56  (0.07-2.02) | 10 | 1.70  (0.81-3.12) | 21 | 1.66  (1.03-2.54) |
| Nephritis, nephrotic syndrome and nephrosis | 9 | 2.85  (1.30-5.40) | 2 | 0.56  (0.07-2.02) | 10 | 1.70  (0.81-3.12) | 21 | 1.66  (1.03-2.54) |
| **External injuries** | 8 | 1.53  (0.66-3.02) | 8 | 1.37  (0.59-2.70) | 6 | 0.63  (0.23-1.37) | 22 | 1.07  (0.67-1.62) |
| Accidents and adverse effects | 6 | 1.42  (0.52-3.09) | 6 | 1.26  (0.46-2.74) | 5 | 0.62  (0.20-1.46) | 17 | 1.00  (0.58-1.60) |
| Suicide and self-inflicted injury | 2 | 2.93  (0.36-10.60) | 2 | 2.75  (0.33-9.93) | 0 | NA | 4 | 1.67  (0.46-4.29) |
| Homicide and legal intervention | 0 | NA | 0 | NA | 1 | 7.29  (0.18-40.64) | 1 | 2.92  (0.07-16.28) |
| **Other cause of death** | 89 | 2.10  (1.68-2.58) | 69 | 1.41  (1.10-1.79) | 122 | 1.39  (1.16-1.66) | 280 | 1.56  (1.39-1.76) |
| Alzheimers (ICD-9 and 10 only) | 8 | 0.98  (0.42-1.92) | 11 | 1.14  (0.57-2.05) | 26 | 1.40  (0.91-2.05) | 45 | 1.24  (0.90-1.65) |
| Diabetes mellitus | 10 | 2.24  (1.07-4.12) | 9 | 1.83  (0.84-3.48) | 12 | 1.62  (0.84-2.82) | 31 | 1.84  (1.25-2.62) |
| Congenital anomalies | 0 | NA | 1 | 6.97  (0.18-38.85) | 0 | NA | 1 | 2.08  (0.05-11.57) |
| Certain conditions originating in perinatal period | 0 | NA | 0 | NA | 0 | NA | 0 | NA |
| Complications of pregnancy, childbirth, puerperium | 0 | NA | 0 | NA | 0 | NA | 0 | NA |
| Symptoms, signs and ill-defifined conditions | 10 | 4.50  (2.16-8.28) | 6 | 2.32  (0.85-5.04) | 4 | 0.87  (0.24-2.23) | 20 | 2.13  (1.30-3.28) |
| Other | 61 | 2.22  (1.70-2.86) | 42 | 1.33  (0.96-1.80) | 80 | 1.41  (1.12-1.75) | 183 | 1.58  (1.36-1.82) |

Additional Table 9: Standardized-mortality ratios following gallbladder cancer diagnosis in white patients.
