# Supplementary material for: Non cancer causes of death after gallbladder cancer diagnosis: a population-based analysis
Source: Sci Rep. 2023 Aug 23;13:13746. doi: 10.1038/s41598-023-40134-4 (PMC10447554; doi:10.1038/s41598-023-40134-4)
Supplement: Supplementary file 10 — Supplementary Table 10. [file 41598_2023_40134_MOESM10_ESM.docx]

| Cause of death | <1 year | | 1-3 years | | >3years | | Total | |
| --- | --- | --- | --- | --- | --- | --- | --- | --- |
|  | Observed | SMR(95%CI) | Observed | SMR(95%CI) | Observed | SMR(95%CI) | Observed | SMR(95%CI) |
| **ALL cause of death** | 655 | 46.30  (42.82-49.98) | 270 | 18.02  (15.94-20.31) | 97 | 3.21  (2.60-3.91) | 1022 | 17.22  (16.18-18.31) |
| **Non-cancer of death** | 25 | 2.31  (1.50-3.41) | 23 | 2.01  (1.27-3.01) | 40 | 1.68  (1.20-2.29) | 88 | 1.91  (1.53-2.35) |
| **Cardiovascular diseases** | 12 | 2.28  ()1.18-3.98 | 10 | 1.82  (0.88-3.36) | 13 | 1.18  (0.63-2.02) | 35 | 1.61  (1.12-2.24) |
| Diseases of heart | 8 | 2.20  (0.95-4.33) | 7 | 1.85  (0.74-3.82) | 9 | 1.20  (0.55-2.28) | 24 | 1.61  (1.03-2.39) |
| Hypertension without heart disease | 1 | 4.03  (0.10-22.46) | 1 | 3.66  (0.09-20.38) | 1 | 1.56  (0.04-8.67) | 3 | 2.58  (0.53-7.53) |
| Aortic aneurysm and dissection | 0 | NA | 0 | NA | 0 | NA | 0 | NA |
| Atherosclerosis | 0 | NA | 0 | NA | 0 | NA | 0 | NA |
| Cerebrovascular diseases | 3 | 2.48  (0.51-7.25) | 2 | 1.59  (0.19-5.76) | 3 | 1.19  (0.25-3.47) | 8 | 1.60  (0.69-3.16) |
| Other diseases of arteries, arterioles, capillaries | 0 | NA | 0 | NA | 0 | NA | 0 | NA |
| **Infectious diseases** | 4 | 4.52  (1.23-11.58) | 2 | 2.15  (0.26-7.78) | 5 | 2.66  (0.86-6.20) | 11 | 2.98  (1.49-5.33) |
| Pneumonia and influenza | 3 | 5.54  (1.14-16.19) | 0 | NA | 4 | 3.37  (0.92-8.64) | 7 | 3.05  (1.23-6.29) |
| Syphilis | 0 | NA | 0 | NA | 0 | NA | 0 | NA |
| Tuberculosis | 0 | NA | 0 | NA | 0 | NA | 0 | NA |
| Septicemia | 1 | 5.33  (0.13-29.69) | 0 | NA | 1 | 2.53  (0.06-14.07) | 2 | 2.55  (0.31-9.23) |
| Other infectious diseases | 0 | NA | 2 | 14.31  (1.73-51.69) | 0 | NA | 2 | 3.78  (0.46-13.67) |
| **Respiratory diseases** | 0 | NA | 1 | 1.65  (0.04-9.20) | 3 | 2.48  (0.51-7.25) | 4 | 1.67  (0.45-4.28) |
| Chronic obstructive pulmonary disease and allied Cond | 0 | NA | 1 | 1.65  (0.04-9.20) | 3 | 2.48  (0.51-7.25) | 4 | 1.67  (0.45-4.28) |
| **Gastrointestinal diseases** | 1 | 6.25  (0.16-34.83) | 1 | 5.94  (0.15-33.11) | 0 | NA | 2 | 3.26  (0.40-11.79) |
| Stomach and duodenal ulcers | 0 | NA | 0 | NA | 0 | NA | 0 | NA |
| Chronic liver disease and cirrhosis | 1 | 7.80  (0.20-43.44) | 1 | 7.33  (0.19-40.82) | 0 | NA | 2 | 4.08  (0.49-14.75) |
| **Renal diseases** | 0 | NA | 0 | NA | 2 | 2.83  (0.34-10.22) | 2 | 1.46  (0.18-5.27) |
| Nephritis, nephrotic syndrome and nephrosis | 0 | NA | 0 | NA | 2 | 2.83  (0.34-10.22) | 2 | 1.46  (0.18-5.27) |
| **External injuries** | 1 | 2.14  (0.05t-11.93) | 0 | NA | 3 | 3.23  (0.67-9.44) | 4 | 2.11  (0.58-5.41) |
| Accidents and adverse effects | 1 | 2.64  (0.07-14.73) | 0 | NA | 3 | 3.83  (0.79-11.18) | 4 | 2.56  (0.70-6.54) |
| Suicide and self-inflicted injury | 0 | NA | 0 | NA | 0 | NA | 0 | NA |
| Homicide and legal intervention | 0 | NA | 0 | NA | 0 | NA | 0 | NA |
| **Other cause of death** | 7 | 2.23  (0.89-4.59) | 9 | 2.62  (1.20-4.97) | 14 | 1.79  (0.98-3.01) | 30 | 2.08  (1.41-2.97) |
| Alzheimers (ICD-9 and 10 only) | 0 | NA | 1 | 2.02  (0.05-11.28) | 5 | 3.69  (1.20-8.61) | 6 | 2.64  (0.97-5.74) |
| Diabetes mellitus | 1 | 1.56  (0.04-8.71) | 2 | 2.93  (0.36-10.59) | 4 | 3.03  (0.83-7.76) | 7 | 2.65  (1.07-5.46) |
| Congenital anomalies | 0 | NA | 0 | NA | 0 | NA | 0 | NA |
| Certain conditions originating in perinatal period | 0 | NA | 0 | NA | 0 | NA | 0 | NA |
| Complications of pregnancy, childbirth, puerperium | 0 | NA | 0 | NA | 0 | NA | 0 | NA |
| Symptoms, signs and ill-defifined conditions | 0 | NA | 0 | NA | 0 | NA | 0 | NA |
| Other | 6 | 3.08  (1.13-6.71) | 6 | 2.82  (1.04-6.15) | 5 | 1.04  (0.34-2.42) | 17 | 1.91  (1.11-3.06) |

Additional Table 10: Standardized-mortality ratios following gallbladder cancer diagnosis in American Indian/Alaska Native Asian or Pacific Islander patients.
