# Supplementary material for: Non cancer causes of death after gallbladder cancer diagnosis: a population-based analysis
Source: Sci Rep. 2023 Aug 23;13:13746. doi: 10.1038/s41598-023-40134-4 (PMC10447554; doi:10.1038/s41598-023-40134-4)
Supplement: Supplementary file 11 — Supplementary Table 11. [file 41598_2023_40134_MOESM11_ESM.docx]

| Cause of death | <1 year | | 1-3 years | | >3years | | Total | |
| --- | --- | --- | --- | --- | --- | --- | --- | --- |
|  | Observed | SMR(95%CI) | Observed | SMR(95%CI) | Observed | SMR(95%CI) | Observed | SMR(95%CI) |
| **ALL cause of death** | 3158 | 28.00  (27.03-28.99) | 1420 | 10.40  (9.87-10.96) | 769 | 2.46  (2.29-2.64) | 5347 | 9.51  (9.26-9.77) |
| **Non-cancer of death** | 209 | 2.37  (2.06-2.71) | 165 | 1.53  (1.31-1.78) | 322 | 1.28  (1.14-1.43) | 696 | 1.55  (1.44-1.67) |
| **Cardiovascular diseases** | 107 | 2.40  (1.97-2.91) | 82 | 1.55  (1.23-1.92) | 128 | 1.13  (0.94-1.34) | 317 | 1.50  (1.34-1.68) |
| Diseases of heart | 82 | 2.48  (1.97-3.08) | 68 | 1.73  (1.34-2.19) | 97 | 1.16  (0.94-1.41) | 247 | 1.58  (1.39-1.79) |
| Hypertension without heart disease | 4 | 3.02  (0.82-7.74) | 5 | 2.95  (0.96-6.88) | 8 | 1.79  (0.77-3.53) | 17 | 2.27  (1.32-3.64) |
| Aortic aneurysm and dissection | 1 | 1.60  (0.04-8.90) | 1 | 1.42  (0.04-7.91) | 1 | 0.78  (0.02-4.35) | 3 | 1.15  (0.24-3.36) |
| Atherosclerosis | 2 | 3.13  (0.38-11.30) | 2 | 2.72  (0.33-9.83) | 4 | 3.22  (0.88-8.25) | 8 | 3.06  (1.32-6.03) |
| Cerebrovascular diseases | 16 | 1.92  (1.10-3.11) | 6 | 0.61  (0.22-1.32) | 18 | 0.85  (0.50-1.34) | 40 | 1.01  (0.72-1.38) |
| Other diseases of arteries, arterioles, capillaries | 2 | 3.97  (0.48-14.34) | 0 | NA | 0 | NA | 2 | 0.84  (0.10-3.02) |
| **Infectious diseases** | 14 | 2.41  (1.32-4.04) | 12 | 1.71  (0.89-2.99) | 26 | 1.70  (1.11-2.49) | 52 | 1.85  (1.38-2.43) |
| Pneumonia and influenza | 2 | 0.60  (0.07-2.17) | 6 | 1.51  (0.55-3.29) | 14 | 1.66  (0.91-2.79) | 22 | 1.40  (0.88-2.12) |
| Syphilis | 0 | NA | 0 | NA | 0 | NA | 0 | NA |
| Tuberculosis | 0 | NA | 0 | NA | 0 | NA | 0 | NA |
| Septicemia | 11 | 6.61  (3.30-11.82) | 4 | 1.98  (0.54-5.06) | 5 | 1.11  (0.36-2.58) | 20 | 2.44  (1.49-3.77) |
| Other infectious diseases | 1 | 1.28  (0.03-7.10) | 2 | 2.07  (0.25-7.47) | 7 | 3.06  (1.23-6.31) | 10 | 2.48  (1.19-4.56) |
| **Respiratory diseases** | 11 | 1.67  (0.83-2.98) | 8 | 1.00  (0.43-1.97) | 21 | 1.12  (0.69-1.71) | 40 | 1.20  (0.86-1.63) |
| Chronic obstructive pulmonary disease and allied Cond | 11 | 1.67  (0.83-2.98) | 8 | 1.00  (0.43-1.97) | 21 | 1.12  (0.69-1.71) | 40 | 1.20  (0.86-1.63) |
| **Gastrointestinal diseases** | 6 | 6.73  (2.47-14.64) | 12 | 11.65  (6.02-20.35) | 6 | 2.75  (1.01-5.99) | 24 | 5.85  (3.75-8.71) |
| Stomach and duodenal ulcers | 0 | NA | 4 | 19.25  (5.25-49.29) | 1 | 2.48  (0.06-13.80) | 5 | 6.30  (2.05-14.70) |
| Chronic liver disease and cirrhosis | 6 | 8.46  (3.10-18.40) | 8 | 9.73  (4.20-19.18) | 5 | 2.82  (0.91-6.57) | 19 | 5.74  (3.46-8.97) |
| **Renal diseases** | 6 | 2.63  (0.97-5.73) | 0 | NA | 11 | 1.71  (0.86-3.07) | 17 | 1.48  (0.86-2.37) |
| Nephritis, nephrotic syndrome and nephrosis | 6 | 2.63  (0.97-5.73) | 0 | NA | 11 | 1.71  (0.86-3.07) | 17 | 1.48  (0.86-2.37) |
| **External injuries** | 7 | 2.28  (0.92-4.70) | 4 | 1.07  (0.29-2.74) | 9 | 0.98  (0.45-1.85) | 20 | 1.25  (0.76-1.93) |
| Accidents and adverse effects | 5 | 2.02  (0.66-4.72) | 3 | 0.98  (0.20-2.87) | 8 | 1.03  (0.44-2.03) | 16 | 1.20  (0.69-1.95) |
| Suicide and self-inflicted injury | 2 | 5.49  (0.67-19.84) | 1 | 2.38  (0.06-13.25) | 0 | NA | 3 | 1.80  (0.37-5.25) |
| Homicide and legal intervention | 0 | NA | 0 | NA | 1 | 5.87  (0.15-32.68) | 1 | 2.86  (0.07-15.91) |
| **Other cause of death** | 58 | 2.29  (1.74-2.97) | 47 | 1.45  (1.07-1.93) | 121 | 1.39  (1.15-1.66) | 226 | 1.56  (1.37-1.78) |
| Alzheimers (ICD-9 and 10 only) | 3 | 0.66  (0.14-1.93) | 5 | 0.84  (0.27-1.95) | 26 | 1.47  (0.96-2.16) | 34 | 1.21  (0.84-1.69) |
| Diabetes mellitus | 8 | 2.35  (1.01-4.62) | 6 | 1.49  (0.55-3.25) | 15 | 1.76  (0.99-2.91) | 29 | 1.82  (1.22-2.61) |
| Congenital anomalies | 0 | NA | 1 | 10.27  (0.26-57.20) | 0 | NA | 1 | 2.62  (0.07-14.61) |
| Certain conditions originating in perinatal period | 0 | NA | 0 | NA | 0 | NA | 0 | NA |
| Complications of pregnancy, childbirth, puerperium | 0 | NA | 0 | NA | 0 | NA | 0 | NA |
| Symptoms, signs and ill-defifined conditions | 7 | 5.08  (2.04-10.46) | 5 | 2.77  (0.90-6.46) | 4 | 0.87  (0.24-2.23) | 16 | 2.06  (1.17-3.34) |
| Other | 40 | 2.52  (1.80-3.43) | 30 | 1.46  (0.99-2.09) | 76 | 1.36  (1.07-1.70) | 146 | 1.58  (1.33-1.86) |

Additional Table 11: Standardized-mortality ratios following gallbladder cancer diagnosis with year of diagnosis between 2000-2009.
