# Supplementary material for: Non cancer causes of death after gallbladder cancer diagnosis: a population-based analysis
Source: Sci Rep. 2023 Aug 23;13:13746. doi: 10.1038/s41598-023-40134-4 (PMC10447554; doi:10.1038/s41598-023-40134-4)
Supplement: Supplementary file 12 — Supplementary Table 12. [file 41598_2023_40134_MOESM12_ESM.docx]

| Cause of death | <1 year | | 1-3 years | | >3years | | Total | |
| --- | --- | --- | --- | --- | --- | --- | --- | --- |
|  | Observed | SMR(95%CI) | Observed | SMR(95%CI) | Observed | SMR(95%CI) | Observed | SMR(95%CI) |
| **ALL cause of death** | 2670 | 28.81  (27.73-29.93) | 1163 | 12.50  (11.79-13.24) | 213 | 3.93  (3.42-4.50) | 4046 | 16.87  (16.35-17.39) |
| **Non-cancer of death** | 146 | 2.02  (1.71-2.38) | 112 | 1.54  (1.27-1.85) | 65 | 1.52  (1.17-1.94) | 323 | 1.72  (1.54-1.92) |
| **Cardiovascular diseases** | 58 | 1.87  (1.42-2.42) | 44 | 1.41  (1.02-1.89) | 22 | 1.21  (0.76-1.84) | 124 | 1.54  (1.28-1.84) |
| Diseases of heart | 42 | 1.83  (1.32-2.47) | 36 | 1.55  (1.09-2.15) | 15 | 1.12  (0.63-1.85) | 93 | 1.56  (1.26-1.91) |
| Hypertension without heart disease | 4 | 3.12  (0.85-7.99) | 1 | 0.77  (0.02-4.28) | 1 | 1.31  (0.03-7.28) | 6 | 1.79  (0.66-3.90) |
| Aortic aneurysm and dissection | 0 | NA | 2 | 5.74  (0.70-20.73) | 0 | NA | 2 | 2.23  (0.27-8.07) |
| Atherosclerosis | 0 | NA | 0 | NA | 0 | NA | 0 | NA |
| Cerebrovascular diseases | 12 | 2.10  (1.09-3.68) | 5 | 0.87  (0.28-2.03) | 5 | 1.46  (0.47-3.41) | 22 | 1.48  (0.93-2.24) |
| Other diseases of arteries, arterioles, capillaries | 0 | NA | 0 | NA | 1 | 4.70  (0.12-26.16) | 1 | 1.06  (0.03-5.91) |
| **Infectious diseases** | 19 | 4.36  (2.63-6.81) | 12 | 2.75  (1.42-4.81) | 5 | 2.01  (0.65-4.69) | 36 | 3.21  (2.25-4.45) |
| Pneumonia and influenza | 6 | 2.77  (1.02-6.03) | 3 | 1.37  (0.28-4.00) | 1 | 0.80  (0.02-4.43) | 10 | 1.78  (0.85-3.28) |
| Syphilis | 0 | NA | 0 | NA | 0 | NA | 0 | NA |
| Tuberculosis | 0 | NA | 0 | NA | 0 | NA | 0 | NA |
| Septicemia | 9 | 6.38  (2.92-12.12) | 5 | 3.54  (1.15-8.27) | 4 | 4.88  (1.33-12.49) | 18 | 4.94  (2.93-7.81) |
| Other infectious diseases | 4 | 5.26  (1.43-13.47) | 4 | 5.42  (1.48-13.87) | 0 | NA | 8 | 4.21  (1.82-8.29) |
| **Respiratory diseases** | 7 | 1.19  (0.48-2.46) | 5 | 0.85  (0.28-1.99) | 3 | 0.86  (0.18-2.52) | 15 | 0.98  (0.55-1.62) |
| Chronic obstructive pulmonary disease and allied Cond | 7 | 1.19  (0.48-2.46) | 5 | 0.85  (0.28-1.99) | 3 | 0.86  (0.18-2.52) | 15 | 0.98  (0.55-1.62) |
| **Gastrointestinal diseases** | 2 | 2.27  (0.28-8.20) | 1 | 1.16  (0.03-6.49) | 0 | NA | 3 | 1.34  (0.28-3.93) |
| Stomach and duodenal ulcers | 0 | NA | 0 | NA | 0 | NA | 0 | NA |
| Chronic liver disease and cirrhosis | 2 | 2.60  (0.32-9.40) | 1 | 1.34  (0.03-7.45) | 0 | NA | 3 | 1.54  (0.32-4.51) |
| **Renal diseases** | 6 | 3.16  (1.16-6.88) | 2 | 1.05  (0.13-3.78) | 2 | 1.84  (0.22-6.63) | 10 | 2.04  (0.98-3.75) |
| Nephritis, nephrotic syndrome and nephrosis | 6 | 3.16  (1.16-6.88) | 2 | 1.05  (0.13-3.78) | 2 | 1.84  (0.22-6.63) | 10 | 2.04  (0.98-3.75) |
| **External injuries** | 4 | 1.27  (0.35-3.26) | 5 | 1.59  (0.52-3.71) | 1 | 0.54  (0.01-3.04) | 10 | 1.23  (0.59-2.26) |
| Accidents and adverse effects | 4 | 1.58  (0.43-4.03) | 4 | 1.57  (0.43-4.01) | 1 | 0.66  (0.02-3.68) | 9 | 1.36  (0.62-2.59) |
| Suicide and self-inflicted injury | 0 | NA | 1 | 2.51  (0.06-14.00) | 0 | NA | 1 | 0.98  (0.02-5.45) |
| Homicide and legal intervention | 0 | NA | 0 | NA | 0 | NA | 0 | NA |
| **Other cause of death** | 50 | 1.99  (1.48-2.63) | 43 | 1.69  (1.22-2.27) | 32 | 2.10  (1.44-2.97) | 125 | 1.90  (1.58-2.26) |
| Alzheimers (ICD-9 and 10 only) | 5 | 1.07  (0.35-2.49) | 7 | 1.43  (0.58-2.96) | 8 | 2.50  (1.08-4.93) | 20 | 1.57  (0.96-2.42) |
| Diabetes mellitus | 6 | 2.21  (0.81-4.80) | 7 | 2.59  (1.04-5.34) | 3 | 1.97  (0.41-5.74) | 16 | 2.30  (1.32-3.74) |
| Congenital anomalies | 0 | NA | 0 | NA | 0 | NA | 0 | NA |
| Certain conditions originating in perinatal period | 0 | NA | 0 | NA | 0 | NA | 0 | NA |
| Complications of pregnancy, childbirth, puerperium | 0 | NA | 0 | NA | 0 | NA | 0 | NA |
| Symptoms, signs and ill-defifined conditions | 3 | 2.49  (0.51-7.29) | 1 | 0.84  (0.02-4.66) | 0 | NA | 4 | 1.33  (0.36-3.39) |
| Other | 36 | 2.19  (1.54-3.03) | 28 | 1.68  (1.12-2.43) | 21 | 2.13  (1.32-3.26) | 85 | 1.98  (1.58-2.45) |

Additional Table 12: Standardized-mortality ratios for following gallbladder cancer diagnosis with year of diagnosis between 2010-2017.
