# Supplementary material for: Non cancer causes of death after gallbladder cancer diagnosis: a population-based analysis
Source: Sci Rep. 2023 Aug 23;13:13746. doi: 10.1038/s41598-023-40134-4 (PMC10447554; doi:10.1038/s41598-023-40134-4)
Supplement: Supplementary file 13 — Supplementary Table 13. [file 41598_2023_40134_MOESM13_ESM.docx]

| Cause of death | <1 year | | 1-3 years | | >3years | | Total | |
| --- | --- | --- | --- | --- | --- | --- | --- | --- |
|  | Observed | SMR(95%CI) | Observed | SMR(95%CI) | Observed | SMR(95%CI) | Observed | SMR(95%CI) |
| **ALL cause of death** | 184 | 5.94  (5.11-6.86) | 188 | 3.85  (3.32-4.45) | 171 | 2.01  (1.72-2.34) | 543 | 3.30  (3.03-3.59) |
| **Non-cancer of death** | 51 | 2.07  (1.54-2.72) | 53 | 1.37  (1.02-1.79) | 96 | 1.40  (1.13-1.71) | 200 | 1.51  (1.31-1.74) |
| **Cardiovascular diseases** | 20 | 1.77  (1.08-2.73) | 26 | 1.49  (0.97-2.18) | 39 | 1.31  (0.93-1.79) | 85 | 1.45  (1.16-1.79) |
| Diseases of heart | 13 | 1.55  (0.82-2.64) | 23 | 1.77  (1.12-2.66) | 26 | 1.19  (0.77-1.74) | 62 | 1.43  (1.10-1.83) |
| Hypertension without heart disease | 0 | NA | 0 | NA | 6 | 4.77  (1.75-10.38) | 6 | 2.54  (0.93-5.53) |
| Aortic aneurysm and dissection | 0 | NA | 0 | NA | 0 | NA | 0 | NA |
| Atherosclerosis | 0 | NA | 0 | NA | 1 | 3.35  (0.08-18.69) | 1 | 1.62  (0.04-9.05) |
| Cerebrovascular diseases | 7 | 3.35  (1.35-6.90) | 3 | 0.93  (0.19-2.71) | 6 | 1.05  (0.39-2.29) | 16 | 1.45  (0.83-2.36) |
| Other diseases of arteries, arterioles, capillaries | 0 | NA | 0 | NA | 0 | NA | 0 | NA |
| **Infectious diseases** | 4 | 2.60  (0.71-6.65) | 5 | 2.08  (0.68-4.86) | 8 | 1.98  (0.86-3.90) | 17 | 2.13  (1.24-3.41) |
| Pneumonia and influenza | 0 | NA | 3 | 2.34  (0.48-6.83) | 6 | 2.78  (1.02-6.04) | 9 | 2.10  (0.96-3.99) |
| Syphilis | 0 | NA | 0 | NA | 0 | NA | 0 | NA |
| Tuberculosis | 0 | NA | 0 | NA | 0 | NA | 0 | NA |
| Septicemia | 2 | 4.36  (0.53-15.76) | 2 | 2.77  (0.33-9.99) | 0 | NA | 4 | 1.66  (0.45-4.24) |
| Other infectious diseases | 2 | 8.44  (1.02-30.49) | 0 | NA | 2 | 3.20  (0.39-11.56) | 4 | 3.22  (0.88-8.24) |
| **Respiratory diseases** | 3 | 1.63  (0.34-4.77) | 4 | 1.37  (0.37-3.50) | 9 | 1.74  (0.80-3.31) | 16 | 1.61  (0.92-2.62) |
| Chronic obstructive pulmonary disease and allied Cond | 3 | 1.63  (0.34-4.77) | 4 | 1.37  (0.37-3.50) | 9 | 1.74  (0.80-3.31) | 16 | 1.61  (0.92-2.62) |
| **Gastrointestinal diseases** | 1 | 4.19  (0.11-23.35) | 0 | NA | 1 | 1.59  (0.04-8.83) | 2 | 1.59  (0.19-5.74) |
| Stomach and duodenal ulcers | 0 | NA | 0 | NA | 0 | NA | 0 | NA |
| Chronic liver disease and cirrhosis | 1 | 5.06  (0.13-28.21) | 0 | NA | 1 | 1.90  (0.05-10.60) | 2 | 1.90  (0.23-6.88) |
| **Renal diseases** | 3 | 4.64  (0.96-13.57) | 0 | NA | 1 | 0.59  (0.01-3.28) | 4 | 1.19  (0.32-3.05) |
| Nephritis, nephrotic syndrome and nephrosis | 3 | 4.64  (0.96-13.57) | 0 | NA | 1 | 0.59  (0.01-3.28) | 4 | 1.19  (0.32-3.05) |
| **External injuries** | 2 | 2.10  (0.25-7.60) | 2 | 1.31  (0.16-4.73) | 5 | 1.91  (0.62-4.46) | 9 | 1.77  (0.81-3.35) |
| Accidents and adverse effects | 2 | 2.55  (0.31-9.23) | 2 | 1.60  (0.19-5.77) | 4 | 1.81  (0.49-4.63) | 8 | 1.88  (0.81-3.71) |
| Suicide and self-inflicted injury | 0 | NA | 0 | NA | 0 | NA | 0 | NA |
| Homicide and legal intervention | 0 | NA | 0 | NA | 1 | 19.79  (0.50-110.27) | 1 | 8.93  (0.23-49.76) |
| **Other cause of death** | 18 | 2.20  (1.30-3.47) | 16 | 1.22  (0.70-1.99) | 33 | 1.34  (0.92-1.88) | 67 | 1.46  (1.13-1.85) |
| Alzheimers (ICD-9 and 10 only) | 2 | 1.24  (0.15-4.47) | 4 | 1.57  (0.43-4.01) | 9 | 1.73  (0.79-3.29) | 15 | 1.60  (0.90-2.24) |
| Diabetes mellitus | 1 | 1.14  (0.03-6.36) | 6 | 4.33  (1.59-9.43) | 3 | 1.32  (0.27-3.86) | 10 | 2.21  (1.06-4.06) |
| Congenital anomalies | 0 | NA | 0 | NA | 0 | NA | 0 | NA |
| Certain conditions originating in perinatal period | 0 | NA | 0 | NA | 0 | NA | 0 | NA |
| Complications of pregnancy, childbirth, puerperium | 0 | NA | 0 | NA | 0 | NA | 0 | NA |
| Symptoms, signs and ill-defifined conditions | 2 | 4.62  (0.56-16.68) | 1 | 1.44  (0.04-8.04) | 1 | 0.80  (0.02-4.46) | 4 | 1.68  (0.46-4.31) |
| Other | 13 | 2.48  (1.32-4.24) | 5 | 0.60  (0.69-1.39) | 20 | 1.26  (0.77-1.94) | 38 | 1.29  (0.91-1.77) |

Additional Table 13: Standardized-mortality ratios following gallbladder cancer diagnosis in localized stage.
