# Supplementary material for: Non cancer causes of death after gallbladder cancer diagnosis: a population-based analysis
Source: Sci Rep. 2023 Aug 23;13:13746. doi: 10.1038/s41598-023-40134-4 (PMC10447554; doi:10.1038/s41598-023-40134-4)
Supplement: Supplementary file 14 — Supplementary Table 14. [file 41598_2023_40134_MOESM14_ESM.docx]

| Cause of death | <1 year | | 1-3 years | | >3years | | Total | |
| --- | --- | --- | --- | --- | --- | --- | --- | --- |
|  | Observed | SMR(95%CI) | Observed | SMR(95%CI) | Observed | SMR(95%CI) | Observed | SMR(95%CI) |
| **ALL cause of death** | 1484 | 16.48  (15.65-17.34) | 1102 | 10.04  (9.45-10.65) | 426 | 3.25  (2.95-3.57) | 3012 | 9.10  (8.78-9.43) |
| **Non-cancer of death** | 140 | 1.98  (1.67-2.34) | 122 | 1.42  (1.18-1.69) | 143 | 1.37  (1.15-1.61) | 405 | 1.55  (1.40-1.71) |
| **Cardiovascular diseases** | 66 | 2.07  (1.60-2.64) | 55 | 1.43  (1.08-1.86) | 54 | 1.18  (0.89-1.55) | 175 | 1.51  (1.29-1.75) |
| Diseases of heart | 48 | 2.03  (1.50-2.69) | 45 | 1.57  (1.15-2.10) | 39 | 1.16  (0.82-1.58) | 132 | 1.53  (1.28-1.82) |
| Hypertension without heart disease | 5 | 4.15  (1.35-9.68) | 2 | 1.33  (0.16-4.82) | 1 | 0.53  (0.01-2.96) | 8 | 1.74  (0.75-3.43) |
| Aortic aneurysm and dissection | 0 | NA | 2 | 4.35  (0.53-15.70) | 0 | NA | 2 | 1.48  (0.18-5.36) |
| Atherosclerosis | 1 | 2.92  (0.07-16.27) | 0 | NA | 2 | 4.58  (0.56-16.56) | 3 | 2.55  (0.53-7.45) |
| Cerebrovascular diseases | 11 | 1.87  (0.93-3.35) | 6 | 0.85  (0.31-1.84) | 11 | 1.29  (0.65-2.31) | 28 | 1.30  (0.87-1.89) |
| Other diseases of arteries, arterioles, capillaries | 1 | 2.71  (0.07-15.09) | 0 | NA | 1 | 1.90  (0.05-10.57) | 2 | 1.49  (0.18-5.37) |
| **Infectious diseases** | 10 | 2.28  (1.09-4.19) | 7 | 1.31  (0.53-2.71) | 11 | 1.77  (0.88-3.16) | 28 | 1.76  (1.17-2.54) |
| Pneumonia and influenza | 3 | 1.30  (0.27-3.81) | 2 | 0.72  (0.09-2.60) | 5 | 1.52  (0.50-3.56) | 10 | 1.20  (0.57-2.20) |
| Syphilis | 0 | NA | 0 | NA | 0 | NA | 0 | NA |
| Tuberculosis | 0 | NA | 0 | NA | 0 | NA | 0 | NA |
| Septicemia | 7 | 5.17  (2.08-10.65) | 3 | 1.81  (0.37-5.28) | 3 | 1.54  (0.32-4.51) | 13 | 2.62  (1.40-4.48) |
| Other infectious diseases | 0 | NA | 2 | 2.32  (0.28-8.38) | 3 | 3.08  (0.63-9.00) | 5 | 1.96  (0.64-4.59) |
| **Respiratory diseases** | 6 | 1.07  (0.39-2.34) | 6 | 0.88  (0.32-1.92) | 8 | 0.99  (0.43-1.95) | 20 | 0.98  (0.60-1.51) |
| Chronic obstructive pulmonary disease and allied Cond | 6 | 1.07  (0.39-2.34) | 6 | 0.88  (0.32-1.92) | 8 | 0.99  (0.43-1.95) | 20 | 0.98  (0.60-1.51) |
| **Gastrointestinal diseases** | 5 | 6.64  (2.16-15.49) | 3 | 3.29  (0.68-9.63) | 2 | 1.99  (0.24-7.29) | 10 | 3.75  (1.80-6.89) |
| Stomach and duodenal ulcers | 0 | NA | 0 | NA | 0 | NA | 0 | NA |
| Chronic liver disease and cirrhosis | 5 | 7.86  (2.55-18.34) | 3 | 3.89  (0.80-11.36) | 2 | 2.37  (0.29-8.54) | 10 | 4.44  (2.13-8.16) |
| **Renal diseases** | 4 | 2.14  (0.58-5.48) | 2 | 0.87  (0.11-3.14) | 7 | 2.58  (1.04-5.31) | 13 | 1.89  (1.01-3.23) |
| Nephritis, nephrotic syndrome and nephrosis | 4 | 2.14  (0.58-5.48) | 2 | 0.87  (0.11-3.14) | 7 | 2.58  (1.04-5.31) | 13 | 1.89  (1.01-3.23) |
| **External injuries** | 6 | 2.16  (0.79-4.71) | 2 | 0.59  (0.07-2.13) | 3 | 0.74  (0.15-2.16) | 11 | 1.08  (0.54-1.92) |
| Accidents and adverse effects | 5 | 2.21  (0.72-5.16) | 1 | 0.36  (0.01-2.01) | 3 | 0.88  (0.18-2.58) | 9 | 1.07  (0.49-2.03) |
| Suicide and self-inflicted injury | 1 | 3.04  (0.08-16.91) | 1 | 2.50  (0.06-13.95) | 0 | NA | 2 | 1.74  (0.21-6.28) |
| Homicide and legal intervention | 0 | NA | 0 | NA | 0 | NA | 0 | NA |
| **Other cause of death** | 43 | 1.84  (1.33-2.47) | 47 | 1.62  (1.19-2.15) | 58 | 1.57  (1.19-2.13) | 148 | 1.66  (1.40-1.95) |
| Alzheimers (ICD-9 and 10 only) | 4 | 0.91  (0.25-2.32) | 7 | 1.27  (0.51-2.61) | 12 | 1.61  (0.83-2.81) | 23 | 1.32  (0.84-1.98) |
| Diabetes mellitus | 7 | 2.66  (1.07-5.49) | 4 | 1.25  (0.34-3.21) | 7 | 1.92  (0.77-3.96) | 18 | 1.90  (1.13-3.01) |
| Congenital anomalies | 0 | NA | 1 | 12.60  (0.32-70.21) | 0 | NA | 1 | 4.29  (0.11-23.92) |
| Certain conditions originating in perinatal period | 0 | NA | 0 | NA | 0 | NA | 0 | NA |
| Complications of pregnancy, childbirth, puerperium | 0 | NA | 0 | NA | 0 | NA | 0 | NA |
| Symptoms, signs and ill-defifined conditions | 4 | 3.32  (0.91-8.51) | 3 | 2.03  (0.42-5.92) | 2 | 1.12  (0.14-4.04) | 9 | 2.01  (0.92-3.82) |
| Other | 28 | 1.85  (1.23-2.68) | 32 | 1.70  (1.17-2.40) | 37 | 1.55  (1.09-2.13) | 97 | 1.68  (1.36-2.05) |

Additional Table 14: Standardized-mortality ratios following gallbladder cancer diagnosis in regional stage.
