# Supplementary material for: Non cancer causes of death after gallbladder cancer diagnosis: a population-based analysis
Source: Sci Rep. 2023 Aug 23;13:13746. doi: 10.1038/s41598-023-40134-4 (PMC10447554; doi:10.1038/s41598-023-40134-4)
Supplement: Supplementary file 15 — Supplementary Table 15. [file 41598_2023_40134_MOESM15_ESM.docx]

| Cause of death | <1 year | | 1-3 years | | >3years | | Total | |
| --- | --- | --- | --- | --- | --- | --- | --- | --- |
|  | Observed | SMR(95%CI) | Observed | SMR(95%CI) | Observed | SMR(95%CI) | Observed | SMR(95%CI) |
| **ALL cause of death** | 2706 | 77.42  (74.53-80.39) | 710 | 48.34  (44.85-52.03) | 56 | 5.58  (4.21-7.24) | 3472 | 58.17  (56.25-60.14) |
| **Non-cancer of death** | 86 | 3.27  (2.61-4.04) | 33 | 2.98  (2.05-4.19) | 8 | 1.00  (0.43-1.97) | 127 | 2.80  (2.33-3.33) |
| **Cardiovascular diseases** | 34 | 2.92  (2.02-4.07) | 12 | 2.49  (1.29-4.35) | 3 | 0.86  (0.18-2.50) | 49 | 2.45  (1.81-3.24) |
| Diseases of heart | 25 | 2.87  (1.86-4.24) | 9 | 2.50  (1.14-4.74) | 3 | 1.16  (0.24-3.39) | 37 | 2.48  (1.75-3.42) |
| Hypertension without heart disease | 3 | 6.86  (1.41-20.04) | 1 | 5.42  (0.14-30.18) | 0 | NA | 4 | 5.19  (1.42-13.30) |
| Aortic aneurysm and dissection | 1 | 6.58  (0.17-36.67) | 1 | 16.61  (0.42-92.54) | 0 | NA | 2 | 8.05  (0.98-29.09) |
| Atherosclerosis | 0 | NA | 0 | NA | 0 | NA | 0 | NA |
| Cerebrovascular diseases | 5 | 2.37  (0.77-5.53) | 1 | 1.15  (0.03-6.38) | 0 | NA | 6 | 1.65  (0.60-3.59) |
| Other diseases of arteries, arterioles, capillaries | 0 | NA | 0 | NA | 0 | NA | 0 | NA |
| **Infectious diseases** | 15 | 8.95  (5.01-14.76) | 5 | 7.24  (2.35-16.89) | 1 | 2.14  (0.05-11.92) | 21 | 7.41  (4.59-11.32) |
| Pneumonia and influenza | 5 | 6.25  (2.03-14.60) | 1 | 3.09  (0.08-17.23) | 0 | NA | 6 | 4.39  (1.61-9.56) |
| Syphilis | 0 | NA | 0 | NA | 0 | NA | 0 | NA |
| Tuberculosis | 0 | NA | 0 | NA | 0 | NA | 0 | NA |
| Septicemia | 8 | 14.55  (6.28-28.67) | 1 | 4.34  (0.11-24.19) | 1 | 6.80  (0.17-37.90) | 10 | 10.79  (5.17-19.84) |
| Other infectious diseases | 2 | 6.30  (0.76-22.74) | 3 | 22.40  (4.62-65.47) | 0 | NA | 5 | 9.50  (3.09-22.18) |
| **Respiratory diseases** | 3 | 1.34  (0.28-3.93) | 0 | NA | 0 | NA | 3 | 0.80  (0.16-2.33) |
| Chronic obstructive pulmonary disease and allied Cond | 3 | 1.34  (0.28-3.93) | 0 | NA | 0 | NA | 3 | 0.80  (0.16-2.33) |
| **Gastrointestinal diseases** | 1 | 2.58  (0.07-14.38) | 1 | 6.02  (0.15-33.53) | 1 | 12.07  (0.31-67.25) | 3 | 4.71  (0.97-13.78) |
| Stomach and duodenal ulcers | 0 | NA | 1 | 54.84  (1.39-305.55) | 1 | 83.62  (2.12-465.91) | 2 | 26.63  (3.22-96.19) |
| Chronic liver disease and cirrhosis | 1 | 2.92  (0.07-16.27) | 0 | NA | 0 | NA | 1 | 1.78  (0.05-9.93) |
| **Renal diseases** | 4 | 5.53  (1.51-14.16) | 0 | NA | 1 | 4.95  (0.13-27.58) | 5 | 4.09  (1.33-9.55) |
| Nephritis, nephrotic syndrome and nephrosis | 4 | 5.53  (1.51-14.16) | 0 | NA | 1 | 4.95  (0.13-27.58) | 5 | 4.09  (1.33-9.55) |
| **External injuries** | 2 | 1.65  (0.20-5.95) | 2 | 3.87  (0.47-13.98) | 0 | NA | 4 | 1.95  (0.53-5.00) |
| Accidents and adverse effects | 1 | 1.05  (0.03-5.88) | 2 | 4.95  (0.60-17.87) | 0 | NA | 3 | 1.86  (0.38-5.43) |
| Suicide and self-inflicted injury | 1 | 5.55  (0.14-30.91) | 0 | NA | 0 | NA | 1 | 3.43  (0.09-19.11) |
| Homicide and legal intervention | 0 | NA | 0 | NA | 0 | NA | 0 | NA |
| **Other cause of death** | 27 | 3.19  (2.10-4.65) | 13 | 3.58  (1.91-6.12) | 2 | 0.70  (0.08-2.52) | 42 | 2.81  (2.02-3.80) |
| Alzheimers (ICD-9 and 10 only) | 0 | NA | 0 | NA | 0 | NA | 0 | NA |
| Diabetes mellitus | 3 | 2.69  (0.56-7.88) | 2 | 4.37  (0.53-15.78) | 0 | NA | 5 | 2.70  (0.88-6.31) |
| Congenital anomalies | 0 | NA | 0 | NA | 0 | NA | 0 | NA |
| Certain conditions originating in perinatal period | 0 | NA | 0 | NA | 0 | NA | 0 | NA |
| Complications of pregnancy, childbirth, puerperium | 0 | NA | 0 | NA | 0 | NA | 0 | NA |
| Symptoms, signs and ill-defifined conditions | 1 | 2.43  (0.06-13.53) | 1 | 5.42  (0.14-30.19) | 0 | NA | 2 | 2.71  (0.33-9.80) |
| Other | 23 | 4.18  (2.65-6.18) | 10 | 4.22  (2.02-7.76) | 2 | 1.08  (0.13-3.92) | 35 | 3.60  (2.51-5.01) |

Additional Table 15: Standardized-mortality ratios following gallbladder cancer diagnosis in distant stage.
