# Supplementary material for: Non cancer causes of death after gallbladder cancer diagnosis: a population-based analysis
Source: Sci Rep. 2023 Aug 23;13:13746. doi: 10.1038/s41598-023-40134-4 (PMC10447554; doi:10.1038/s41598-023-40134-4)
Supplement: Supplementary file 16 — Supplementary Table 16. [file 41598_2023_40134_MOESM16_ESM.docx]

| Cause of death | <1 year | | 1-3 years | | >3years | | Total | |
| --- | --- | --- | --- | --- | --- | --- | --- | --- |
|  | Observed | SMR(95%CI) | Observed | SMR(95%CI) | Observed | SMR(95%CI) | Observed | SMR(95%CI) |
| **ALL cause of death** | 1539 | 14.81  (14.08-15.57) | 1252 | 8.98  (8.49-9.50) | 616 | 2.59  (2.39-2.80) | 3407 | 7.08  (6.84-7.32) |
| **Non-cancer of death** | 144 | 1.77  (1.49-2.09) | 178 | 1.62  (1.39-1.88) | 242 | 1.27  (1.11-1.44) | 564 | 1.48  (1.36-1.60) |
| **Cardiovascular diseases** | 68 | 1.78  (1.39-2.26) | 82 | 1.60  (1.28-1.99) | 100 | 1.17  (0.96-1.43) | 250 | 1.43  (1.26-1.62) |
| Diseases of heart | 51 | 1.80  (1.34-2.37) | 66 | 1.74  (1.35-2.21) | 77 | 1.22  (0.97-1.53) | 194 | 1.50  (1.30-1.73) |
| Hypertension without heart disease | 4 | 3.02  (0.82-7.73) | 4 | 2.19  (0.60-5.60) | 5 | 1.47  (0.48-3.43) | 13 | 1.98  (1.06-3.39) |
| Aortic aneurysm and dissection | 1 | 2.03  (0.05-11.32) | 2 | 3.13  (0.38-11.32) | 0 | NA | 3 | 1.43  (0.30-4.18) |
| Atherosclerosis | 1 | 2.18  (0.06-12.17) | 2 | 3.27  (0.40-11.81) | 2 | 2.24  (0.27-8.10) | 5 | 2.55  (0.83-5.95) |
| Cerebrovascular diseases | 9 | 1.27  (0.58-2.40) | 8 | 0.84  (0.36-1.66) | 16 | 1.00  (0.57-1.62) | 33 | 1.01  (0.70-1.42) |
| Other diseases of arteries, arterioles, capillaries | 2 | 4.57  (0.55-16.52) | 0 | NA | 0 | NA | 2 | 1.01  (0.12-3.64) |
| **Infectious diseases** | 13 | 2.53  (1.34-4.32) | 11 | 1.59  (0.79-2.85) | 19 | 1.64  (0.99-2.57) | 43 | 1.82  (1.32-2.45) |
| Pneumonia and influenza | 4 | 1.43  (0.39-3.67) | 3 | 0.80  (0.16-2.33) | 12 | 1.90  (0.98-3.31) | 19 | 1.47  (0.89-2.30) |
| Syphilis | 0 | NA | 0 | NA | 0 | NA | 0 | NA |
| Tuberculosis | 0 | NA | 0 | NA | 0 | NA | 0 | NA |
| Septicemia | 7 | 4.52  (1.82-9.32) | 5 | 2.41  (0.78-5.62) | 4 | 1.17  (0.32-2.99) | 16 | 2.27  (1.30-3.68) |
| Other infectious diseases | 2 | 2.57  (0.31-9.30) | 3 | 2.90  (0.60-8.48) | 3 | 1.73  (0.36-5.05) | 8 | 2.25  (0.97-4.44) |
| **Respiratory diseases** | 9 | 1.42  (0.65-2.69) | 10 | 1.19  (0.57-2.19) | 11 | 0.76  (0.38-1.36) | 30 | 1.03  (0.69-1.47) |
| Chronic obstructive pulmonary disease and allied Cond | 9 | 1.42  (0.65-2.69) | 10 | 1.19  (0.57-2.19) | 11 | 0.76  (0.38-1.36) | 30 | 1.03  (0.69-1.47) |
| **Gastrointestinal diseases** | 3 | 3.41  (0.70-9.96) | 7 | 6.07  (2.44-12.50) | 4 | 2.30  (0.63-5.89) | 14 | 3.71  (2.03-6.23) |
| Stomach and duodenal ulcers | 0 | NA | 4 | 20.56  (5.60-52.64) | 0 | NA | 4 | 6.15  (1.68-15.75) |
| Chronic liver disease and cirrhosis | 3 | 4.10  (0.85-11.98) | 3 | 3.13  (0.64-9.14) | 4 | 2.80  (0.76-7.16) | 10 | 3.20  (1.54-5.89) |
| **Renal diseases** | 6 | 2.84  (1.04-6.18) | 2 | 0.70  (0.08-2.53) | 7 | 1,44  (0.58-2.97) | 15 | 1.53  (0.85-2.52) |
| Nephritis, nephrotic syndrome and nephrosis | 6 | 2.84  (1.04-6.18) | 2 | 0.70  (0.08-2.53) | 7 | 1,44  (0.58-2.97) | 15 | 1.53  (0.85-2.52) |
| **External injuries** | 5 | 1.60  (0.52-3.74) | 5 | 1.20  (0.39-2.79) | 7 | 0.97  (0.39-2.00) | 17 | 1.17  (0.68-1.88) |
| Accidents and adverse effects | 5 | 1.97  (0.64-4.61) | 4 | 1.17  (0.32-3.01) | 7 | 1.16  (0.46-2.38) | 16 | 1.33  (0.76-2.17) |
| Suicide and self-inflicted injury | 0 | NA | 1 | 2.01  (0.05-11.22) | 0 | NA | 1 | 0.63  (0.02-3.49) |
| Homicide and legal intervention | 0 | NA | 0 | NA | 0 | NA | 0 | NA |
| **Other cause of death** | 40 | 1.56  (1.11-2.12) | 61 | 1.73  (1.33-2.23) | 94 | 1.42  (1.15-1.73) | 195 | 1.53  (1.33-1.76) |
| Alzheimers (ICD-9 and 10 only) | 5 | 1.05  (0.34-2.46) | 9 | 1.36  (0.62-2.58) | 18 | 1.33  (0.79-2.10) | 32 | 1.29  (0.88-1.82) |
| Diabetes mellitus | 4 | 1.30  (0.35-3.33) | 10 | 2.45  (1.18-4.51) | 11 | 1.69  (0.84-3.02) | 25 | 1.83  (1.18-2.70) |
| Congenital anomalies | 0 | NA | 1 | 9.65  (0.24-53.75) | 0 | NA | 1 | 2.96  (0.07-16.50) |
| Certain conditions originating in perinatal period | 0 | NA | 0 | NA | 0 | NA | 0 | NA |
| Complications of pregnancy, childbirth, puerperium | 0 | NA | 0 | NA | 0 | NA | 0 | NA |
| Symptoms, signs and ill-defifined conditions | 5 | 3.80  (1.23-8.86) | 3 | 1.64  (0.34-4.80) | 2 | 0.59  (0.07-2.15) | 10 | 1.54  (0.74-2.83) |
| Other | 26 | 1.58  (1.03-2.31) | 38 | 1.69  (1.19-2.31) | 63 | 1.47  (1.13-1.89) | 127 | 1.55  (1.29-1.85) |

Additional Table 16: Standardized-mortality ratios following gallbladder cancer diagnosis in grade I+II.
