# Supplementary material for: Non cancer causes of death after gallbladder cancer diagnosis: a population-based analysis
Source: Sci Rep. 2023 Aug 23;13:13746. doi: 10.1038/s41598-023-40134-4 (PMC10447554; doi:10.1038/s41598-023-40134-4)
Supplement: Supplementary file 17 — Supplementary Table 17. [file 41598_2023_40134_MOESM17_ESM.docx]

| Cause of death | <1 year | | 1-3 years | | >3years | | Total | |
| --- | --- | --- | --- | --- | --- | --- | --- | --- |
|  | Observed | SMR(95%CI) | Observed | SMR(95%CI) | Observed | SMR(95%CI) | Observed | SMR(95%CI) |
| **ALL cause of death** | 2034 | 36.05  (34.50-37.65) | 762 | 14.44  (13.43-15.50) | 218 | 3.18  (2.77-3.63) | 3014 | 16.96  (16.36-17.58) |
| **Non-cancer of death** | 115 | 2.62  (2.17-3.15) | 46 | 1.11  (0.81-1.48) | 78 | 1.41  (1.12-1.76) | 239 | 1.70  (1.49-1.93) |
| **Cardiovascular diseases** | 52 | 2.52  (1.88-3.30) | 23 | 1.19  (0.76-1.79) | 28 | 1.13  (0.75-1.64) | 103 | 1.59  (1.30-1.93) |
| Diseases of heart | 41 | 2.67  (1.92-3.63) | 19 | 1.33  (0.80-2.07) | 18 | 0.98  (0.58-1.55) | 78 | 1.63  (1.29-2.03) |
| Hypertension without heart disease | 2 | 2.85  (0.34-10.29) | 1 | 1.48  (0.04-8.25) | 1 | 1.03  (0.03-5.74) | 4 | 1.70  (0.46-4.36) |
| Aortic aneurysm and dissection | 0 | NA | 1 | 4.17  (0.11-23.23) | 1 | 3.68  (0.09-20.48) | 2 | 2.56  (0.31-9.24) |
| Atherosclerosis | 0 | NA | 0 | NA | 2 | 7.28  (0.88-26.29) | 2 | 2.63  (0.32-9.52) |
| Cerebrovascular diseases | 9 | 2.34  (1.07-4.44) | 2 | 0.56  (0.07-2.01) | 5 | 1.08  (0.35-2.52) | 16 | 1.32  (0.76-2.15) |
| Other diseases of arteries, arterioles, capillaries | 0 | NA | 0 | NA | 1 | 3.49  (0.09-19.43) | 1 | 1.34  (0.03-7.44) |
| **Infectious diseases** | 11 | 3.96  (1.98-7.09) | 7 | 2.70  (1.09-5.57) | 6 | 1.81  (0.67-3.95) | 24 | 2.77  (1.77-4.12) |
| Pneumonia and influenza | 3 | 2.01  (0.42-5.88) | 3 | 2.15  (0.44-6.29) | 2 | 2.11  (0.13-4.02) | 8 | 1.71  (0.74-3.37) |
| Syphilis | 0 | NA | 0 | NA | 0 | NA | 0 | NA |
| Tuberculosis | 0 | NA | 0 | NA | 0 | NA | 0 | NA |
| Septicemia | 8 | 9.42  (4.07-18.56) | 1 | 1.26  (0.03-6.99) | 1 | 0.99  (0.03-5.52) | 10 | 3.77  (1.81-6.93) |
| Other infectious diseases | 0 | NA | 3 | 7.78  (1.60-22.72) | 3 | 6.10  (1.26-17.84) | 6 | 4.62  (1.70-10.06) |
| **Respiratory diseases** | 6 | 1.74  (0.64-3.79) | 2 | 0.62  (0.07-2.22) | 5 | 1.20  (0.39-2.81) | 13 | 1.20  (0.64-2.05) |
| Chronic obstructive pulmonary disease and allied Cond | 6 | 1.74  (0.64-3.79) | 2 | 0.62  (0.07-2.22) | 5 | 1.20  (0.39-2.81) | 13 | 1.20  (0.64-2.05) |
| **Gastrointestinal diseases** | 1 | 2.02  (0.05-11.26) | 2 | 4.71  (0.57-17.03) | 0 | NA | 3 | 2.15  (0.44-6.29) |
| Stomach and duodenal ulcers | 0 | NA | 0 | NA | 0 | NA | 0 | NA |
| Chronic liver disease and cirrhosis | 1 | 2.42  (0.06-13.46) | 2 | 5.69  (0.69-20.56) | 0 | NA | 3 | 2.60  (0.54-7.60) |
| **Renal diseases** | 4 | 3.51  (0.96-8.98) | 0 | NA | 5 | 3.54  (1.15-8.26) | 9 | 2.48  (1.13-4.70) |
| Nephritis, nephrotic syndrome and nephrosis | 4 | 3.51  (0.96-8.98) | 0 | NA | 5 | 3.54  (1.15-8.26) | 9 | 2.48  (1.13-4.70) |
| **External injuries** | 1 | 0.59  (0.01-3.28) | 2 | 4.71  (0.57-17.03) | 1 | 0.50  (0.01-2.81) | 4 | 0.76  (0.21-1.96) |
| Accidents and adverse effects | 1 | 0.73  (0.02-4.07) | 1 | 0.78  (0.02-4.37) | 1 | 0.60  (0.02-3.32) | 3 | 0.69  (0.14-2.03) |
| Suicide and self-inflicted injury | 0 | NA | 1 | 5.65  (0.14-31.50) | 0 | NA | 1 | 1.75  (0.04-9.73) |
| Homicide and legal intervention | 0 | NA | 0 | NA | 0 | NA | 0 | NA |
| **Other cause of death** | 40 | 2.92  (2.09-3.98) | 10 | 0.75  (0.36-1.38) | 33 | 1.72  (1.19-2.42) | 83 | 1.80  (1.43-2.23) |
| Alzheimers (ICD-9 and 10 only) | 2 | 0.81  (0.10-2.92) | 1 | 0.40  (0.01-2.22) | 9 | 2.27  (1.04-4.31) | 12 | 1.34  (0.69-2.34) |
| Diabetes mellitus | 7 | 4.12  (1.65-8.48) | 0 | NA | 3 | 1.63  (0.34-4.75) | 10 | 1.97  (0.94-3.62) |
| Congenital anomalies | 0 | NA | 0 | NA | 0 | NA | 0 | NA |
| Certain conditions originating in perinatal period | 0 | NA | 0 | NA | 0 | NA | 0 | NA |
| Complications of pregnancy, childbirth, puerperium | 0 | NA | 0 | NA | 0 | NA | 0 | NA |
| Symptoms, signs and ill-defifined conditions | 3 | 4.34  (0.89-12.68) | 1 | 1.48  (0.04-8.26) | 1 | 1.00  (0.03-5.59) | 5 | 2.12  (0.69-4.94) |
| Other | 28 | 3.19  (2.12-4.61) | 8 | 0.93  (0.40-1.84) | 20 | 1.63  (0.99-2.51) | 56 | 1.89  (1.43-2.45) |

Additional Table 17: Standardized-mortality ratios following gallbladder cancer diagnosis in grade III+IV.
