# Supplementary material for: Non cancer causes of death after gallbladder cancer diagnosis: a population-based analysis
Source: Sci Rep. 2023 Aug 23;13:13746. doi: 10.1038/s41598-023-40134-4 (PMC10447554; doi:10.1038/s41598-023-40134-4)
Supplement: Supplementary file 18 — Supplementary Table 18. [file 41598_2023_40134_MOESM18_ESM.docx]

| Cause of death | <1 year | | 1-3 years | | >3years | | Total | |
| --- | --- | --- | --- | --- | --- | --- | --- | --- |
|  | Observed | SMR(95%CI) | Observed | SMR(95%CI) | Observed | SMR(95%CI) | Observed | SMR(95%CI) |
| **ALL cause of death** | 3123 | 18.62  (17.97-19.28) | 2047 | 9.68  (9.26-10.11) | 921 | 2.66  (2.49-2.83) | 6091 | 8.39  (8.18-8.61) |
| **Non-cancer of death** | 256 | 1.95  (1.72-2.21) | 243 | 1.46  (1.28-1.66) | 358 | 1.29  (1.16-1.43) | 857 | 1.49  (1.39-1.59) |
| **Cardiovascular diseases** | 120 | 1.94  (1.61-2.32) | 113 | 1.46  (1.20-1.75) | 140 | 1.13  (0.95-1.33) | 373 | 1.42  (1.28-1.57) |
| Diseases of heart | 91 | 1.98  (1.60-2.44) | 92 | 1.60  (1.29-1.96) | 104 | 1.14  (0.93-1.38) | 287 | 1.47  (1.31-1.65) |
| Hypertension without heart disease | 5 | 2.36  (0.76-5.50) | 5 | 1.81  (0.59-4.23) | 8 | 1.62  (0.70-3.20) | 18 | 1.84  (1.09-2.90) |
| Aortic aneurysm and dissection | 1 | 1.25  (0.03-6.97) | 3 | 3.10  (0.64-9.05) | 1 | 0.72  (0.02-3.99) | 5 | 1.58  (0.51-3.69) |
| Atherosclerosis | 2 | 2.64  (0.32-9.55) | 2 | 2.14  (0.26-7.72) | 4 | 3.05  (0.83-7.81) | 8 | 2.66  (1.15-5.25) |
| Cerebrovascular diseases | 19 | 1.65  (0.99-2.57) | 11 | 0.76  (0.38-1.36) | 22 | 0.95  (0.59-1.43) | 52 | 1.06  (0.79-1.39) |
| Other diseases of arteries, arterioles, capillaries | 2 | 2.82  (0.34-10.17) | 0 | NA | 1 | 0.71  (0.02-3.95) | 3 | 0.99  (0.21-2.91) |
| **Infectious diseases** | 20 | 2.41  (1.47-3.72) | 18 | 1.72  (1.02-2.72) | 27 | 1.61  (1.06-2.34) | 65 | 1.83  (1.41-2.33) |
| Pneumonia and influenza | 5 | 1.11  (0.36-2.59) | 7 | 1.23  (0.50-2.54) | 14 | 1.53  (0.84-2.57) | 26 | 1.35  (0.88-1.97) |
| Syphilis | 0 | NA | 0 | NA | 0 | NA | 0 | NA |
| Tuberculosis | 0 | NA | 0 | NA | 0 | NA | 0 | NA |
| Septicemia | 13 | 5.19  (2.77-8.88) | 7 | 2.21  (0.89-4.56) | 6 | 1.19  (0.44-2.59) | 26 | 2.43  (1.59-3.56) |
| Other infectious diseases | 2 | 1.61  (0.19-5.80) | 4 | 2.55  (0.70-6.54) | 7 | 2.76  (1.11-5.68) | 13 | 2.43  (1.29-4.15) |
| **Respiratory diseases** | 16 | 1.57  (0.90-2.55) | 13 | 1.02  (0.54-1.74) | 22 | 1.04  (0.65-1.58) | 51 | 1.16  (0.86-1.52) |
| Chronic obstructive pulmonary disease and allied Cond | 16 | 1.57  (0.90-2.55) | 13 | 1.02  (0.54-1.74) | 22 | 1.04  (0.65-1.58) | 51 | 1.16  (0.86-1.52) |
| **Gastrointestinal diseases** | 4 | 2.81  (0.77-7.19) | 10 | 5.77  (2.76-10.60) | 5 | 1.96  (0.64-4.58) | 19 | 3.33  (2.01-5.20) |
| Stomach and duodenal ulcers | 0 | NA | 4 | 13.59  (3.70-34.79) | 0 | NA | 4 | 4.09  (1.11-10.46) |
| Chronic liver disease and cirrhosis | 4 | 3.38  (0.92-8.66) | 6 | 4.17  (1.53-9.07) | 5 | 2.38  (0.77-5.55) | 15 | 3.17  (1.78-5.24) |
| **Renal diseases** | 11 | 3.24  (1.62-5.79) | 2 | 0.46  (0.06-1.67) | 12 | 1.69  (0.88-2.96) | 25 | 1.69  (1.09-2.49) |
| Nephritis, nephrotic syndrome and nephrosis | 11 | 3.24  (1.62-5.79) | 2 | 0.46  (0.06-1.67) | 12 | 1.69  (0.88-2.96) | 25 | 1.69  (1.09-2.49) |
| **External injuries** | 7 | 1.39  (0.56-2.87) | 6 | 0.95  (0.35-2.06) | 10 | 0.96  (0.46-1.76) | 23 | 1.06  (0.67-1.58) |
| Accidents and adverse effects | 6 | 1.48  (0.54-3.21) | 5 | 0.97  (0.31-2.26) | 9 | 1.03  (0.47-1.95) | 20 | 1.11  (0.68-1.72) |
| Suicide and self-inflicted injury | 1 | 1.63  (0.04-9.11) | 1 | 1.34  (0.03-7.44) | 0 | NA | 2 | 0.83  (0.10-2.99) |
| Homicide and legal intervention | 0 | NA | 0 | NA | 1 | 5.07  (0.13-28.22) | 1 | 2.07  (0.05-11.52) |
| **Other cause of death** | 78 | 1.89  (1.50-2.36) | 81 | 1.52  (1.21-1.89) | 142 | 1.47  (1.24-1.74) | 301 | 1.58  (1.40-1.77) |
| Alzheimers (ICD-9 and 10 only) | 7 | 0.92  (0.37-1.90) | 11 | 1.10  (0.55-1.96) | 30 | 1.53  (1.03-2.18) | 48 | 1.29  (0.95-1.71) |
| Diabetes mellitus | 11 | 2.21  (1.10-3.96) | 10 | 1.62  (0.78-2.98) | 17 | 1.79  (1.04-2.87) | 38 | 1.84  (1.30-2.53) |
| Congenital anomalies | 0 | NA | 1 | 6.38  (0.16-35.56) | 0 | NA | 1 | 1.95  (0.05-10.84) |
| Certain conditions originating in perinatal period | 0 | NA | 0 | NA | 0 | NA | 0 | NA |
| Complications of pregnancy, childbirth, puerperium | 0 | NA | 0 | NA | 0 | NA | 0 | NA |
| Symptoms, signs and ill-defifined conditions | 9 | 4.25  (1.94-8.07) | 5 | 1.81  (0.59-4.22) | 4 | 0.82  (0.22-2.09) | 18 | 1.84  (1.09-2.91) |
| Other | 51 | 1.93  (1.44-2.54) | 54 | 1.58  (1.19-2.06) | 91 | 1.47  (1.18-1.80) | 196 | 1.60  (1.38-1.84) |

Additional Table 18: Standardized-mortality ratios following gallbladder cancer diagnosis in patients who received surgery.
