# Supplementary material for: Non cancer causes of death after gallbladder cancer diagnosis: a population-based analysis
Source: Sci Rep. 2023 Aug 23;13:13746. doi: 10.1038/s41598-023-40134-4 (PMC10447554; doi:10.1038/s41598-023-40134-4)
Supplement: Supplementary file 19 — Supplementary Table 19. [file 41598_2023_40134_MOESM19_ESM.docx]

| Cause of death | <1 year | | 1-3 years | | >3years | | Total | |
| --- | --- | --- | --- | --- | --- | --- | --- | --- |
|  | Observed | SMR(95%CI) | Observed | SMR(95%CI) | Observed | SMR(95%CI) | Observed | SMR(95%CI) |
| **ALL cause of death** | 2672 | 72.26  (69.54-75.05) | 529 | 30.97  (28.39-33.73) | 56 | 2.84  (2.14-3.69) | 3257 | 44.14  (42.64-45.69) |
| **Non-cancer of death** | 99 | 3.45  (2.81-4.21) | 34 | 2.54  (1.76-3.56) | 27 | 1.67  (1.10-2.43) | 160 | 2.75  (2.34-3.21) |
| **Cardiovascular diseases** | 45 | 3.37  (2.46-4.50) | 13 | 2.10  (1.12-3.60) | 9 | 1.25  (0.57-2.37) | 67 | 2.50  (1.94-3.18) |
| Diseases of heart | 33 | 3.31  (2.28-4.65) | 12 | 2.61  (1.35-4.55) | 8 | 1.50  (0.65-2.95) | 53 | 2.66  (1.99-3.48) |
| Hypertension without heart disease | 3 | 6.36  (1.31-18.57) | 1 | 4.49  (0.11-25.00) | 0 | NA | 4 | 4.05  (1.10-10.38) |
| Aortic aneurysm and dissection | 0 | NA | 0 | NA | 0 | NA | 0 | NA |
| Atherosclerosis | 0 | NA | 0 | NA | 0 | NA | 0 | NA |
| Cerebrovascular diseases | 9 | 3.66  (1.67-6.95) | 0 | NA | 1 | 0.75  (0.02-4.16) | 10 | 2.03  (0.97-3.73) |
| Other diseases of arteries, arterioles, capillaries | 0 | NA | 0 | NA | 0 | NA | 0 | NA |
| **Infectious diseases** | 13 | 7.09  (3.77-12.12) | 6 | 7.07  (2.59-15.39) | 4 | 4.18  (1.14-10.70) | 23 | 6.32  (4.01-9.48) |
| Pneumonia and influenza | 3 | 3.09  (0.64-9.04) | 2 | 4.39  (0.53-15.86) | 1 | 1.91  (0.05-10.64) | 6 | 3.08  (1.13-6.70) |
| Syphilis | 0 | NA | 0 | NA | 0 | NA | 0 | NA |
| Tuberculosis | 0 | NA | 0 | NA | 0 | NA | 0 | NA |
| Septicemia | 7 | 12.53  (5.04-25.82) | 2 | 7.83  (0.95-28.28) | 3 | 10.55  (2.18-30.84) | 12 | 10.92  (5.64-19.08) |
| Other infectious diseases | 3 | 10.18  (2.10-29.76) | 2 | 15.09  (1.83-54.52) | 0 | NA | 5 | 8.74  (2.84-20.40) |
| **Respiratory diseases** | 2 | 0.89  (0.11-3.23) | 0 | NA | 2 | 1.74  (0.21-6.30) | 4 | 0.90  (0.25-2.31) |
| Chronic obstructive pulmonary disease and allied Cond | 2 | 0.89  (0.11-3.23) | 0 | NA | 2 | 1.74  (0.21-6.30) | 4 | 0.90  (0.25-2.31) |
| **Gastrointestinal diseases** | 4 | 11.65  (3.17-29.83) | 3 | 20.15  (4.15-58.88) | 1 | 8.28  (0.21-46.11) | 8 | 13.05  (5.63-25.71) |
| Stomach and duodenal ulcers | 0 | NA | 0 | NA | 1 | 41.22  (1.04-229.65) | 1 | 9.96  (0.25-55.48) |
| Chronic liver disease and cirrhosis | 4 | 13.74  (3.74-35.19) | 3 | 23.99  (4.95-70.11) | 0 | NA | 7 | 13.65  (5.49-28.13) |
| **Renal diseases** | 1 | 1.31  (0.03-7.30) | 0 | NA | 1 | 2.44  (0.06-13.61) | 2 | 1.31  (0.16-4.73) |
| Nephritis, nephrotic syndrome and nephrosis | 1 | 1.31  (0.03-7.30) | 0 | NA | 1 | 2.44  (0.06-13.61) | 2 | 1.31  (0.16-4.73) |
| **External injuries** | 4 | 3.42  (0.93-8.76) | 3 | 5.61  (1.16-16.41) | 0 | NA | 7 | 3.05  (1.23-6.28) |
| Accidents and adverse effects | 3 | 3.22  (0.66-9.42) | 2 | 4.65  (0.56-16.81) | 0 | NA | 5 | 2.68  (0.87-6.25) |
| Suicide and self-inflicted injury | 1 | 6.43  (0.16-35.81) | 1 | 14.85  (0.38-82.73) | 0 | NA | 2 | 7.34  (0.89-26.53) |
| Homicide and legal intervention | 0 | NA | 0 | NA | 0 | NA | 0 | NA |
| **Other cause of death** | 30 | 3.34  (2.25-4.77) | 9 | 2.11  (0.96-4.00) | 10 | 1.73  (0.83-3.18) | 49 | 2.58  (1.91-3.40) |
| Alzheimers (ICD-9 and 10 only) | 1 | 0.63  (0.02-3.52) | 1 | 1.29  (0.03-7.19) | 4 | 3.24  (0.88-8.29) | 6 | 1.67  (0.61-3.63) |
| Diabetes mellitus | 3 | 2.64  (0.54-7.71) | 3 | 5.86  (1.21-17.13) | 1 | 1.96  (0.05-10.94) | 7 | 3.24  (1.30-6.68) |
| Congenital anomalies | 0 | NA | 0 | NA | 0 | NA | 0 | NA |
| Certain conditions originating in perinatal period | 0 | NA | 0 | NA | 0 | NA | 0 | NA |
| Complications of pregnancy, childbirth, puerperium | 0 | NA | 0 | NA | 0 | NA | 0 | NA |
| Symptoms, signs and ill-defifined conditions | 1 | 2.20  (0.06-12.26) | 1 | 4.55  (0.12-25.33) | 0 | NA | 2 | 2.05  (0.25-7.40) |
| Other | 25 | 4.33  (2.80-6.39) | 4 | 1.45  (0.40-3.72) | 5 | 1.35  (0.44-3.14) | 34 | 2.78  (1.92-3.88) |

Additional Table 19: Standardized-mortality ratios following gallbladder cancer diagnosis in patients without surgery.
