# Supplementary material for: Non cancer causes of death after gallbladder cancer diagnosis: a population-based analysis
Source: Sci Rep. 2023 Aug 23;13:13746. doi: 10.1038/s41598-023-40134-4 (PMC10447554; doi:10.1038/s41598-023-40134-4)
Supplement: Supplementary file 20 — Supplementary Table 20. [file 41598_2023_40134_MOESM20_ESM.docx]

| Cause of death | <1 year | | 1-3 years | | >3years | | Total | |
| --- | --- | --- | --- | --- | --- | --- | --- | --- |
|  | Observed | SMR(95%CI) | Observed | SMR(95%CI) | Observed | SMR(95%CI) | Observed | SMR(95%CI) |
| **ALL cause of death** | 621 | 23.18  (21.39-25.08) | 607 | 20.48  (18.89-22.18) | 190 | 3.78  (3.26-4.35) | 1418 | 13.29  (12.60-14.00) |
| **Non-cancer of death** | 33 | 1.68  (1.15-2.35) | 34 | 1.55  (1.07-2.17) | 47 | 1.20  (0.88-1.60) | 114 | 1.41  (1.17-1.70) |
| **Cardiovascular diseases** | 13 | 1.43  (0.76-2.44) | 10 | 1.00  (0.48-1.83) | 21 | 1.22  (0.76-1.87) | 44 | 1.21  (0.88-1.63) |
| Diseases of heart | 8 | 1.17  (0.51-2.31) | 9 | 1.20  (0.55-2.27) | 15 | 1.18  (0.66-1.95) | 32 | 1.18  (0.81-1.67) |
| Hypertension without heart disease | 3 | 9.90  (2.04-28.93) | 1 | 2.84  (0.07-15.84) | 1 | 1.45  (0.04-8.10) | 5 | 3.72  (1.21-8.69) |
| Aortic aneurysm and dissection | 0 | NA | 0 | NA | 0 | NA | 0 | NA |
| Atherosclerosis | 0 | NA | 0 | NA | 0 | NA | 0 | NA |
| Cerebrovascular diseases | 2 | 1.22  (0.15-4.39) | 0 | NA | 5 | 1.55  (0.50-3.62) | 7 | 1.05  (0.42-2.16) |
| Other diseases of arteries, arterioles, capillaries | 0 | NA | 0 | NA | 0 | NA | 0 | NA |
| **Infectious diseases** | 2 | 1.56  (0.19-5.62) | 3 | 2.09  (0.43-6.11) | 6 | 2.48  (0.91-5.41) | 11 | 2.14  (1.07-3.83) |
| Pneumonia and influenza | 0 | NA | 1 | 1.47  (0.04-8.20) | 2 | 1.62  (0.20-5.87) | 3 | 1.19  (0.25-3.47) |
| Syphilis | 0 | NA | 0 | NA | 0 | NA | 0 | NA |
| Tuberculosis | 0 | NA | 0 | NA | 0 | NA | 0 | NA |
| Septicemia | 2 | 4.75  (0.57-17.14) | 1 | 2.10  (0.05-11.72) | 1 | 1.33  (0.03-7.39) | 4 | 2.42  (0.66-6.20) |
| Other infectious diseases | 0 | NA | 1 | 3.70  (0.09-20.60) | 3 | 7.19  (1.48-21.01) | 4 | 4.30  (1.17-11.00) |
| **Respiratory diseases** | 1 | 0.58  (0.01-3.24) | 3 | 1.60  (0.33-4.68) | 3 | 0.95  (0.20-2.77) | 7 | 1.04  (0.42-2.13) |
| Chronic obstructive pulmonary disease and allied Cond | 1 | 0.58  (0.01-3.24) | 3 | 1.60  (0.33-4.68) | 3 | 0.95  (0.20-2.77) | 7 | 1.04  (0.42-2.13) |
| **Gastrointestinal diseases** | 0 | NA | 6 | 17.46  (6.41-38.01) | 2 | 4.24  (0.51-15.32) | 8 | 7.07  (3.05-13.93) |
| Stomach and duodenal ulcers | 0 | NA | 4 | 98.50  (26.84-252.19) | 0 | NA | 4 | 27.90  (7.60-71.44) |
| Chronic liver disease and cirrhosis | 0 | NA | 2 | 6.60  (0.80-23.85) | 2 | 4.92  (0.60-17.76) | 4 | 4.05  (1.10-10.36) |
| **Renal diseases** | 0 | NA | 0 | NA | 1 | 0.98  (0.02-5.44) | 1 | 0.46  (0.01-2.55) |
| Nephritis, nephrotic syndrome and nephrosis | 0 | NA | 0 | NA | 1 | 0.98  (0.02-5.44) | 1 | 0.46  (0.01-2.55) |
| **External injuries** | 2 | 2.17  (0.26-7.85) | 2 | 2.00  (0.24-7.21) | 1 | 0.62  (0.02-3.45) | 5 | 1.41  (0.46-3.30) |
| Accidents and adverse effects | 1 | 1.43  (0.04-7.95) | 1 | 1.30  (0.03-7.23) | 1 | 0.76  (0.02-4.24) | 3 | 1.08  (0.22-3.15) |
| Suicide and self-inflicted injury | 1 | 6.72  (0.17-37.44) | 1 | 6.42  (0.16-35.75) | 0 | NA | 2 | 3.99  (0.48-14.42) |
| Homicide and legal intervention | 0 | NA | 0 | NA | 0 | NA | 0 | NA |
| **Other cause of death** | 15 | 2.57  (1.44-4.24) | 10 | 1.51  (0.72-2.77) | 13 | 0.98  (0.52-1.68) | 38 | 1.48  (1.05-2.03) |
| Alzheimers (ICD-9 and 10 only) | 0 | NA | 0 | NA | 2 | 0.80  (0.10-2.91) | 2 | 0.47  (0.06-1.68) |
| Diabetes mellitus | 1 | 1.10  (0.03-6.11) | 0 | NA | 1 | 0.67  (0.02-3.72) | 2 | 0.59  (0.07-2.12) |
| Congenital anomalies | 0 | NA | 0 | NA | 0 | NA | 0 | NA |
| Certain conditions originating in perinatal period | 0 | NA | 0 | NA | 0 | NA | 0 | NA |
| Complications of pregnancy, childbirth, puerperium | 0 | NA | 0 | NA | 0 | NA | 0 | NA |
| Symptoms, signs and ill-defifined conditions | 1 | 3.65  (0.09-20.36) | 2 | 6.33  (0.77-22.85) | 0 | NA | 3 | 2.43  (0.50-7.09) |
| Other | 13 | 3.44  (1.83-5.88) | 8 | 1.86  (0.80-3.66) | 10 | 1.16  (0.56-2.14) | 31 | 1.86  (1.26-2.64) |

Additional Table 20: Standardized-mortality ratios following gallbladder cancer diagnosis in patients who received radiotherapy.
