# Supplementary material for: Non cancer causes of death after gallbladder cancer diagnosis: a population-based analysis
Source: Sci Rep. 2023 Aug 23;13:13746. doi: 10.1038/s41598-023-40134-4 (PMC10447554; doi:10.1038/s41598-023-40134-4)
Supplement: Supplementary file 21 — Supplementary Table 21. [file 41598_2023_40134_MOESM21_ESM.docx]

| Cause of death | <1 year | | 1-3 years | | >3years | | Total | |
| --- | --- | --- | --- | --- | --- | --- | --- | --- |
|  | Observed | SMR(95%CI) | Observed | SMR(95%CI) | Observed | SMR(95%CI) | Observed | SMR(95%CI) |
| **ALL cause of death** | 5207 | 29.14  (28.36-29.94) | 1976 | 9.88  (9.45-10.33) | 792 | 2.50  (2.33-2.68) | 7975 | 11.47  (11.22-11.73) |
| **Non-cancer of death** | 322 | 2.29  (2.04-2.55) | 243 | 1.53  (1.35-1.74) | 340 | 1.33  (1.19-1.48) | 905 | 1.63  (1.53-1.74) |
| **Cardiovascular diseases** | 152 | 2.29  (1.94-2.69) | 116 | 1.56  (1.29-1.88) | 129 | 1.13  (0.94-1.34) | 397 | 1.56  (1.41-1.72) |
| Diseases of heart | 116 | 2.36  (1.95-2.83) | 95 | 1.73  (1.40-2.11) | 97 | 1.15  (0.93-1.40) | 308 | 1.63  (1.46-1.83) |
| Hypertension without heart disease | 5 | 2.17  (0.71-5.07) | 5 | 1.89  (0.61-4.41) | 8 | 1.76  (0.76-3.47) | 18 | 1.90  (1.12-3.00) |
| Aortic aneurysm and dissection | 1 | 1.19  (0.03-6.64) | 3 | 3.30  (0.68-9.63) | 1 | 0.79  (0.02-4.40) | 5 | 1.66  (0.54-3.87) |
| Atherosclerosis | 2 | 2.44  (0.30-8.81) | 2 | 2.20  (0.27-7.96) | 4 | 3.27  (0.89-8.38) | 8 | 2.71  (1.17-5.34) |
| Cerebrovascular diseases | 26 | 2.10  (1.37-3.07) | 11 | 0.79  (0.40-1.42) | 18 | 0.84  (0.50-1.33) | 55 | 1.15  (0.87-1.50) |
| Other diseases of arteries, arterioles, capillaries | 2 | 2.63  (0.32-9.50) | 0 | NA | 1 | 0.77  (0.02-4.30) | 3 | 1.03  (0.21-3.02) |
| **Infectious diseases** | 31 | 3.49  (2.37-4.95) | 21 | 2.11  (1.31-3.23) | 25 | 1.63  (1.05-2.40) | 77 | 2.25  (1.78-2.81) |
| Pneumonia and influenza | 8 | 1.64  (0.71-3.23) | 8 | 1.46  (0.63-2.87) | 13 | 1.54  (0.82-2.63) | 29 | 1.54  (1.03-2.21) |
| Syphilis | 0 | NA | 0 | NA | 0 | NA | 0 | NA |
| Tuberculosis | 0 | NA | 0 | NA | 0 | NA | 0 | NA |
| Septicemia | 18 | 6.78  (4.02-10.72) | 8 | 2.70  (1.17-5.33) | 8 | 1.75  (0.75-3.44) | 34 | 3.34  (2.31-4.66) |
| Other infectious diseases | 5 | 3.84  (1.25-8.96) | 5 | 3.49  (1.13-8.13) | 4 | 1.76  (0.48-4.51) | 14 | 2.80  (1.53-4.69) |
| **Respiratory diseases** | 17 | 1.58  (0.92-2.53) | 10 | 0.83  (0.40-1.53) | 21 | 1.10  (0.68-1.68) | 48 | 1.15  (0.85-1.52) |
| Chronic obstructive pulmonary disease and allied Cond | 17 | 1.58  (0.92-2.53) | 10 | 0.83  (0.40-1.53) | 21 | 1.10  (0.68-1.68) | 48 | 1.15  (0.85-1.52) |
| **Gastrointestinal diseases** | 8 | 5.50  (2.37-10.83) | 7 | 4.53  (1.82-9.33) | 4 | 1.82  (0.50-4.66) | 19 | 3.65  (2.20-5.70) |
| Stomach and duodenal ulcers | 0 | NA | 0 | NA | 1 | 2.48  (0.06-13.79) | 1 | 1.06  (0.03-5.93) |
| Chronic liver disease and cirrhosis | 8 | 6.67  (2.88-13.14) | 7 | 5.53  (2.22-11.39) | 3 | 1.67  (0.34-4.88) | 18 | 4.22  (2.50-6.67) |
| **Renal diseases** | 12 | 3.31  (1.71-5.78) | 2 | 0.49  (0.06-1.77) | 12 | 1.85  (0.96-3.23) | 26 | 1.83  (1.20-2.68) |
| Nephritis, nephrotic syndrome and nephrosis | 12 | 3.31  (1.71-5.78) | 2 | 0.49  (0.06-1.77) | 12 | 1.85  (0.96-3.23) | 26 | 1.83  (1.20-2.68) |
| **External injuries** | 9 | 1.70  (0.78-3.23) | 7 | 1.19  (0.48-2.45) | 9 | 0.95  (0.44-1.81) | 25 | 1.21  (0.78-1.79) |
| Accidents and adverse effects | 8 | 1.86  (0.80-3.66) | 6 | 1.24  (0.46-2.70) | 8 | 1.00  (0.43-1.98) | 22 | 1.28  (0.81-1.95) |
| Suicide and self-inflicted injury | 1 | 1.61  (0.04-8.98) | 1 | 1.51  (0.04-8.41) | 0 | NA | 2 | 0.91  (0.11-3.30) |
| Homicide and legal intervention | 0 | NA | 0 | NA | 1 | 5.88  (0.15-32.77) | 1 | 2.28  (0.06-12.71) |
| **Other cause of death** | 93 | 2.09  (1.68-2.56) | 80 | 1.56  (1.24-1.94) | 140 | 1.57  (1.32-1.86) | 313 | 1.69  (1.51-1.89) |
| Alzheimers (ICD-9 and 10 only) | 8 | 0.95  (0.41-1.88) | 12 | 1.21  (0.63-2.12) | 32 | 1.74  (1.19-2.46) | 52 | 1.42  (1.06-1.86) |
| Diabetes mellitus | 13 | 2.49  (1.33-4.26) | 13 | 2.28  (1.21-3.89) | 17 | 1.99  (1.16-3.19) | 43 | 2.21  (1.60-2.98) |
| Congenital anomalies | 0 | NA | 1 | 7.08  (0.18-39.44) | 0 | NA | 1 | 2.11  (0.05-11.75) |
| Certain conditions originating in perinatal period | 0 | NA | 0 | NA | 0 | NA | 0 | NA |
| Complications of pregnancy, childbirth, puerperium | 0 | NA | 0 | NA | 0 | NA | 0 | NA |
| Symptoms, signs and ill-defifined conditions | 9 | 3.90  (1.78-7.40) | 4 | 1.49  (0.41-3.81) | 4 | 0.87  (0.24-2.24) | 17 | 1.78  (1.04-2.85) |
| Other | 63 | 2.21  (1.70-2.83) | 50 | 1.52  (1.13-2.01) | 87 | 1.52  (1.22-1.87) | 200 | 1.69  (1.46-1.94) |

Additional Table 21: Standardized-mortality ratios following gallbladder cancer diagnosis in patients without radiotherapy.
