# Supplementary material for: Non cancer causes of death after gallbladder cancer diagnosis: a population-based analysis
Source: Sci Rep. 2023 Aug 23;13:13746. doi: 10.1038/s41598-023-40134-4 (PMC10447554; doi:10.1038/s41598-023-40134-4)
Supplement: Supplementary file 22 — Supplementary Table 22. [file 41598_2023_40134_MOESM22_ESM.docx]

| Cause of death | <1 year | | 1-3 years | | >3years | | Total | |
| --- | --- | --- | --- | --- | --- | --- | --- | --- |
|  | Observed | SMR(95%CI) | Observed | SMR(95%CI) | Observed | SMR(95%CI) | Observed | SMR(95%CI) |
| **ALL cause of death** | 2309 | 44.78  (42.98-46.65) | 1274 | 29.55  (27.95-31.22) | 255 | 4.62  (4.07-5.23) | 3838 | 25.62  (24.81-26.44) |
| **Non-cancer of death** | 79 | 2.12  (1.68-2.64) | 49 | 1.56  (1.16-2.07) | 45 | 1.07  (0.78-1.43) | 173 | 1.56  (1.34-1.81) |
| **Cardiovascular diseases** | 34 | 2.04  (1.42-2.86) | 14 | 1.00  (0.55-1.68) | 18 | 0.97  (0.58-1.54) | 66 | 1.34  (1.04-1.71) |
| Diseases of heart | 26 | 2.08  (1.36-3.05) | 13 | 1.24  (0.66-2.12) | 14 | 1.02  (0.56-1.71) | 53 | 1.44  (1.08-1.89) |
| Hypertension without heart disease | 2 | 3.51  (0.43-12.70) | 0 | NA | 0 | NA | 2 | 1.12  (0.14-4.03) |
| Aortic aneurysm and dissection | 0 | NA | 0 | NA | 0 | NA | 0 | NA |
| Atherosclerosis | 0 | NA | 0 | NA | 1 | 5.73  (0.15-31.93) | 1 | 2.21  (0.06-12.31) |
| Cerebrovascular diseases | 6 | 2.03  (0.74-4.41) | 1 | 0.40  (0.01-2.24) | 3 | 0.87  (0.18-2.55) | 10 | 1.13  (0.54-2.07) |
| Other diseases of arteries, arterioles, capillaries | 0 | NA | 0 | NA | 0 | NA | 0 | NA |
| **Infectious diseases** | 11 | 4.56  (2.28-8.16) | 4 | 1.97  (0.54-5.05) | 4 | 1.53  (0.42-3.92) | 19 | 2.69  (1.62-4.21) |
| Pneumonia and influenza | 3 | 2.77  (0.57-8.10) | 1 | 1.08  (0.03-6.02) | 0 | NA | 4 | 1.21  (0.33-3.10) |
| Syphilis | 0 | NA | 0 | NA | 0 | NA | 0 | NA |
| Tuberculosis | 0 | NA | 0 | NA | 0 | NA | 0 | NA |
| Septicemia | 6 | 7.33  (2.69-15.95) | 1 | 1.45  (0.04-8.10) | 2 | 2.37  (0.29-8.56) | 9 | 3.83  (1.75-7.26) |
| Other infectious diseases | 2 | 4.04  (0.49-14.58) | 2 | 4.96  (0.60-17.92) | 2 | 4.41  (0.53-15.94) | 6 | 4.44  (1.63-9.66) |
| **Respiratory diseases** | 5 | 1.46  (0.47-3.40) | 3 | 1.06  (0.22-3.08) | 2 | 0.56  (0.07-2.02) | 10 | 1.02  (0.49-1.87) |
| Chronic obstructive pulmonary disease and allied Cond | 5 | 1.46  (0.47-3.40) | 3 | 1.06  (0.22-3.08) | 2 | 0.56  (0.07-2.02) | 10 | 1.02  (0.49-1.87) |
| **Gastrointestinal diseases** | 0 | NA | 6 | 10.85  (3.98-23.62) | 3 | 5.49  (1.13-16.03) | 9 | 5.05  (2.31-9.59) |
| Stomach and duodenal ulcers | 0 | NA | 4 | 69.55  (18.95-178.08) | 0 | NA | 4 | 20.25  (5.52-51.84) |
| Chronic liver disease and cirrhosis | 0 | NA | 2 | 4.04  (0.49-14.59) | 3 | 6.29  (1.30-18.39) | 5 | 3.16  (1.03-7.37) |
| **Renal diseases** | 1 | 0.97  (0.02-5.42) | 0 | NA | 1 | 0.89  (0.02-4.98) | 2 | 0.66  (0.08-2.40) |
| Nephritis, nephrotic syndrome and nephrosis | 1 | 0.97  (0.02-5.42) | 0 | NA | 1 | 0.89  (0.02-4.98) | 2 | 0.66  (0.08-2.40) |
| **External injuries** | 3 | 1.57  (0.32-4.60) | 4 | 2.56  (0.70-6.56) | 1 | 0.56  (0.01-3.09) | 8 | 1.52  (0.66-2.99) |
| Accidents and adverse effects | 2 | 1.39  (0.17-5.03) | 3 | 2.53  (0.52-7.39) | 1 | 0.69  (0.02-3.85) | 6 | 1.47  (0.54-3.21) |
| Suicide and self-inflicted injury | 1 | 3.06  (0.08-17.05) | 1 | 3.83  (0.10-21.32) | 0 | NA | 2 | 2.42  (0.29-8.75) |
| Homicide and legal intervention | 0 | NA | 0 | NA | 0 | NA | 0 | NA |
| **Other cause of death** | 25 | 2.23  (1.44-3.29) | 18 | 1.88  (1.11-2.97) | 16 | 1.13  (0.65-1.83) | 59 | 1.69  (1.28-2.18) |
| Alzheimers (ICD-9 and 10 only) | 1 | 0.65  (0.02-3.63) | 0 | NA | 1 | 0.39  (0.01-2.18) | 2 | 0.37  (0.04-1.33) |
| Diabetes mellitus | 2 | 1.13  (0.14-4.09) | 2 | 1.36  (0.16-4.92) | 2 | 1.19  (0.14-4.30) | 6 | 1.22  (0.45-2.66) |
| Congenital anomalies | 0 | NA | 0 | NA | 0 | NA | 0 | NA |
| Certain conditions originating in perinatal period | 0 | NA | 0 | NA | 0 | NA | 0 | NA |
| Complications of pregnancy, childbirth, puerperium | 0 | NA | 0 | NA | 0 | NA | 0 | NA |
| Symptoms, signs and ill-defifined conditions | 1 | 1.98  (0.05-11.03) | 2 | 4.60  (0.56-16.62) | 0 | NA | 3 | 1.86  (0.38-5.43) |
| Other | 21 | 2.85  (1.77-4.36) | 14 | 2.23  (1.22-3.74) | 13 | 1.41  (0.75-2.41) | 48 | 2.10  (1.55-2.79) |

Additional Table 22: Standardized-mortality ratios following gallbladder cancer diagnosis in patients who received chemotherapy.
