# Supplementary material for: Non cancer causes of death after gallbladder cancer diagnosis: a population-based analysis
Source: Sci Rep. 2023 Aug 23;13:13746. doi: 10.1038/s41598-023-40134-4 (PMC10447554; doi:10.1038/s41598-023-40134-4)
Supplement: Supplementary file 23 — Supplementary Table 23. [file 41598_2023_40134_MOESM23_ESM.docx]

| Cause of death | <1 year | | 1-3 years | | >3years | | Total | |
| --- | --- | --- | --- | --- | --- | --- | --- | --- |
|  | Observed | SMR(95%CI) | Observed | SMR(95%CI) | Observed | SMR(95%CI) | Observed | SMR(95%CI) |
| **ALL cause of death** | 3519 | 22.86  (22.12-23.63) | 1309 | 7.02  (6.65-7.41) | 727 | 2.33  (2.17-2.51) | 5555 | 8.52  (8.30-8.74) |
| **Non-cancer of death** | 276 | 2.24  (1.98-2.52) | 228 | 1.53  (1.34-1.74) | 342 | 1.36  (1.22-1.51) | 846 | 1.61  (1.51-1.73) |
| **Cardiovascular diseases** | 131 | 2.23  (1.86-2.64) | 112 | 1.59  (1.31-1.92) | 132 | 1.17  (0.98-1.39) | 375 | 1.55  (1.40-1.72) |
| Diseases of heart | 98 | 2.25  (1.83-2.74) | 91 | 1.75  (1.41-2.15) | 98 | 1.18  (0.95-1.43) | 287 | 1.60  (1.42-1.80) |
| Hypertension without heart disease | 6 | 2.95  (1.08-6.41) | 6 | 2.40  (0.88-5.22) | 9 | 2.00  (0.91-3.79) | 21 | 2.32  (1.44-3.55) |
| Aortic aneurysm and dissection | 1 | 1.37  (0.03-7.66) | 3 | 3.52  (0.73-10.30) | 1 | 0.80  (0.02-4.47) | 5 | 1.77  (0.57-4.13) |
| Atherosclerosis | 2 | 2.62  (0.32-9.47) | 2 | 2.26  (0.27-8.18) | 3 | 2.47  (0.51-7.21) | 7 | 2.45  (0.98-5.04) |
| Cerebrovascular diseases | 22 | 1.98  (1.24-3.00) | 10 | 0.76  (0.36-1.40) | 20 | 0.94  (0.58-1.46) | 52 | 1.14  (0.85-1.50) |
| Other diseases of arteries, arterioles, capillaries | 2 | 2.99  (0.36-10.81) | 4 | 3.07  (0.84-7.87) | 5 | 2.24  (0.73-5.22) | 3 | 1.09  (0.23-3.19) |
| **Infectious diseases** | 22 | 2.83  (1.78-4.29) | 20 | 2.14  (1.31-3.31) | 27 | 1.78  (1.17-2.59) | 69 | 2.14  (1.66-2.71) |
| Pneumonia and influenza | 5 | 1.13  (0.37-2.64) | 8 | 1.53  (0.66-3.01) | 15 | 1.79  (1.00-2.96) | 28 | 1.55  (1.03-2.24) |
| Syphilis | 0 | NA | 0 | NA | 0 | NA | 0 | NA |
| Tuberculosis | 0 | NA | 0 | NA | 0 | NA | 0 | NA |
| Septicemia | 14 | 6.21  (3.39-10.41) | 8 | 2.91  (1.26-5.74) | 7 | 1.56  (0.63-3.21) | 29 | 3.06  (2.05-4.39) |
| Other infectious diseases | 3 | 2.86  (0.59-8.36) | 4 | 3.07  (0.84-7.87) | 5 | 2.24  (0.73-5.22) | 12 | 2.62  (1.35-4.57) |
| **Respiratory diseases** | 13 | 1.44  (0.77-2.46) | 10 | 0.91  (0.43-1.67) | 22 | 1.18  (0.74-1.78) | 45 | 1.16  (0.85-1.55) |
| Chronic obstructive pulmonary disease and allied Cond | 13 | 1.44  (0.77-2.46) | 10 | 0.91  (0.43-1.67) | 22 | 1.18  (0.74-1.78) | 45 | 1.16  (0.85-1.55) |
| **Gastrointestinal diseases** | 8 | 7.33  (3.17-14.45) | 7 | 5.24  (2.11-10.79) | 3 | 1.41  (0.29-4.13) | 18 | 3.95  (2.34-6.25) |
| Stomach and duodenal ulcers | 0 | NA | 0 | NA | 1 | 2.51  (0.06-13.98) | 1 | 1.13  (0.03-6.30) |
| Chronic liver disease and cirrhosis | 8 | 9.23  (3.99-18.19) | 7 | 6.52  (2.62-13.43) | 2 | 1.16  (0.14-4.18) | 17 | 4.64  (2.70-7.42) |
| **Renal diseases** | 11 | 3.49  (1.74-6.25) | 2 | 0.52  (0.06-1.88) | 12 | 1.88  (0.97-3.28) | 25 | 1.87  (1.21-2.76) |
| Nephritis, nephrotic syndrome and nephrosis | 11 | 3.49  (1.74-6.25) | 2 | 0.52  (0.06-1.88) | 12 | 1.88  (0.97-3.28) | 25 | 1.87  (1.21-2.76) |
| **External injuries** | 8 | 1.86  (0.80-3.66) | 5 | 0.94  (0.31-2.19) | 9 | 0.97  (0.44-1.85) | 22 | 1.17  (0.73-1.76) |
| Accidents and adverse effects | 7 | 1.96  (0.79-4.03) | 4 | 0.91  (0.25-2.32) | 8 | 1.02  (0.44-2.01) | 19 | 1.20  (0.72-1.87) |
| Suicide and self-inflicted injury | 1 | 2.26  (0.06-12.59) | 1 | 1.80  (0.05-10.00) | 0 | NA | 2 | 1.07  (0.13-3.87) |
| Homicide and legal intervention | 0 | NA | 0 | NA | 1 | 6.11  (0.15-34.04) | 1 | 2.69  (0.07-14.99) |
| **Other cause of death** | 83 | 2.12  (1.69-2.63) | 72 | 1.49  (1.17-1.88) | 137 | 1.56  (1.31-1.84) | 292 | 1.66  (1.48-1.87) |
| Alzheimers (ICD-9 and 10 only) | 7 | 0.91  (0.37-1.88) | 12 | 1.26  (0.65-2.20) | 33 | 1.80  (1.24-2.53) | 52 | 1.46  (1.09-1.92) |
| Diabetes mellitus | 12 | 2.75  (1.42-4.80) | 11 | 2.10  (1.05-3.75) | 16 | 1.92  (1.10-3.11) | 39 | 2.17  (1.54-2.97) |
| Congenital anomalies | 0 | NA | 1 | 8.05  (0.20-44.87) | 0 | NA | 1 | 2.38  (0.06-13.25) |
| Certain conditions originating in perinatal period | 0 | NA | 0 | NA | 0 | NA | 0 | NA |
| Complications of pregnancy, childbirth, puerperium | 0 | NA | 0 | NA | 0 | NA | 0 | NA |
| Symptoms, signs and ill-defifined conditions | 9 | 4.33  (1.98-8.23) | 4 | 1.56  (0.42-3.99) | 4 | 0.88  (0.24-2.25) | 17 | 1.85  (1.08-2.96) |
| Other | 55 | 2.21  (1.66-2.87) | 44 | 1.43  (1.04-1.91) | 84 | 1.48  (1.18-1.84) | 183 | 1.63  (1.40-1.88) |

Additional Table 23: Standardized-mortality ratios following gallbladder cancer diagnosis in patients without chemotherapy.
